# Aggrecan protects against plaque accumulation and is essential for proper microglial responses to plaques

**DOI:** 10.1016/j.celrep.2025.116064

**Published:** 2025-07-31

**Authors:** Rocio A. Barahona, Nellie E. Kwang, Aashna R. Kono-Soosaipillai, Giovanna Rubio Salgado, Kristine M. Tran, Yueh-Hao Lu, Siddharth Reddy, Celia da Cunha, Eric Velazquez-Rivera, Joshua D. Crapser, Xiangmin Xu, Lindsay A. Hohsfield, Kim N. Green

**Affiliations:** 1 Department of Neurobiology and Behavior, Charlie Dunlop School of Biological Sciences, University of California, Irvine, Irvine, CA 92697, USA; 2 Institute for Memory Impairments and Neurological Disorders (UCI MIND), University of California, Irvine, Irvine, CA 92697, USA; 3 Department of Anatomy and Neurobiology, School of Medicine, University of California, Irvine, Irvine, CA 92697, USA; 4 Department of Neurology and Neurological Sciences, Stanford University School of Medicine, Stanford, CA 94305, USA; 5 Lead contact

## Abstract

Alzheimer’s disease (AD) is a neurodegenerative disease characterized by amyloid plaques and neurofibrillary tangles. Recent evidence implicates extracellular matrix (ECM) dysfunction in disease pathogenesis, including extensive loss of perineuronal nets (PNNs). PNNs are neuron-ensheathing condensed ECM structures composed of chondroitin sulfate proteoglycans, including the main constituent aggrecan (ACAN). To explore the role of PNNs in AD, we utilize the 5xFAD model and genetically target *Acan* in *Nestin*-expressing cells, resulting in loss of ACAN and ablation of the PNN structure. In 5xFAD mice, ACAN cKO results in increased plaque deposition, reduced plaque sphericity, and impaired microglia-plaque association. Single-cell spatial transcriptomics identifies an enhanced disease-associated microglia (DAM) phenotype in 5xFAD ACAN cKO mice, which is accompanied by decreased dystrophic neurite formation. Collectively, our data suggest that PNNs may play a crucial role in mediating the microglial response to plaques.

## In brief

Barahona et al. show that loss of aggrecan, a key structural protein in perineuronal nets, increases plaque pathology in mice. Their findings indicate that the lack of aggrecan impairs microglial engagement with plaques and dampens neuritic dystrophy, highlighting a role for the extracellular matrix in neurodegeneration.

## Graphical Abstract

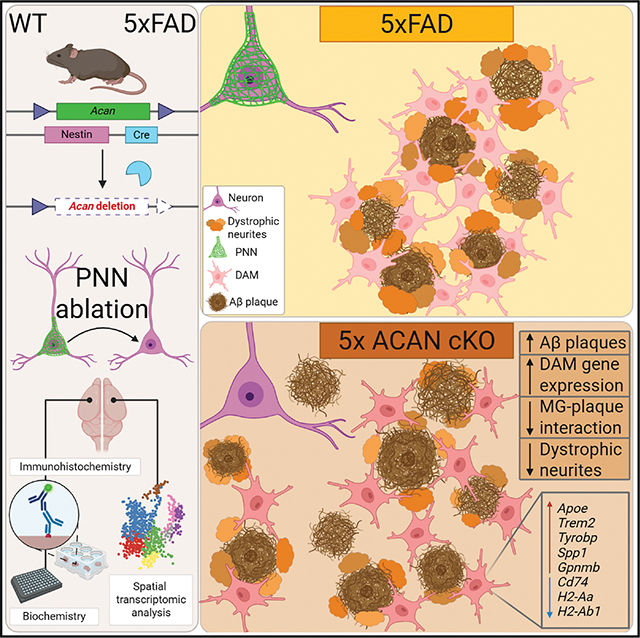

## INTRODUCTION

Alzheimer’s disease (AD) is a progressive neurodegenerative disease characterized by the deposition of aggregated amyloid-beta (Aβ) extracellular plaques and hyperphosphorylated tau intracellular neurofibrillary tangles. Other disease pathological hallmarks include extensive synaptic and neuronal loss, which ultimately lead to memory deficits and cognitive impairment.^1,2^ While amyloid plaques have been hypothesized by many researchers to be the causative factor in AD pathogenesis,^3^ the mechanisms underlying plaque formation in the context of AD remain unclear. Genome-wide association studies have identified genetic loci that contribute to the risk of late-onset AD and show that several of these genes are associated with myeloid cell biology and function (e.g., *TREM2*, *ABCA7*, *PLCG2*, *ABI3*), implicating myeloid cells in AD pathogenesis.^4–7^ However, unresolved questions remain about the biological processes involved in myeloid cell dysfunction and eventual disease progression and neurodegeneration. While accumulating evidence implicates a critical role of microglia in plaque formation and maintenance,^8–10^ our lab recently identified another key role of microglia as important regulators of the extracellular matrix (ECM) during AD.^11^

In the central nervous system (CNS), the ECM is classified into three distinct compartments: (1) the basement membrane underlying blood vessel endothelial cells and glial endfeet, (2) the interstitial matrix serving as both a cellular scaffold and medium for the diffusion of signaling molecules, and (3) the pericellular condensed matrix—known as perineuronal nets (PNNs) —where ECM components directly interact with cell surface receptors to influence various cellular functions while also stabilizing the synapses they enwrap.^12^ The key structural components of PNNs are chondroitin sulfate proteoglycans (CSPGs) of the lectican family (e.g., aggrecan [ACAN], brevican, neurocan, versican), which consist of glycosaminoglycan (GAG) chains covalently attached to a core protein.^13–16^ PNNs are further stabilized by hyaluronan, a non-sulfated GAG that forms the backbone of the net, and link proteins such as hyaluronan and proteoglycan link protein 1 (HAPLN1), which anchor CSPGs to hyaluronan strands and are critical for maintaining net integrity.^17–20^ Additionally, Tenascin-R (TNR), a large glycoprotein of the tenascin family, serves as a crosslinker within the PNN by binding to both CSPGs and cell adhesion molecules, further contributing to the compact and functionally restrictive architecture of the net.^20,21^ PNNs are typically identified histologically using markers that label distinct components of the PNN structure: (1) antibodies targeting the protein core of the principal CSPG ACAN (encoded by the *Acan* gene) and (2) *Wisteria floribunda* agglutinin (WFA), a plant lectin that recognizes the CS-GAG chains of PNNs.

PNNs ensheathe the soma and proximal synapses of different neuronal subsets across various brain regions but are primarily associated with inhibitory neurons in the cortex.^22–24^ In the healthy adult brain, the ECM influences cell-matrix adhesion, migration, signaling, and proliferation,^25–27^ with PNNs having known roles in restricting plasticity^23,28–31^ and conferring neuroprotection against oxidative stress,^20,32^ aggregated tau protein^33^ and Aβ protein neurotoxicity in cultured cortical neurons.^34^ However, PNN deficits have been reported in a variety of neurological and neuropsychiatric disorders, including adult-onset leukoencephalopathy with axonal spheroids and pigmented glia, multiple sclerosis, traumatic brain injury, epilepsy, schizophrenia, Huntington’s disease, and AD,^11,35–40^ underscoring the relevance of studying PNNs in neurodegeneration and their potential as therapeutic targets.

Recent studies indicate that PNN loss may be implicated in dysregulated synaptic plasticity and neuronal excitatory inhibitory imbalance.^41,42^ While others have noted PNN disruptions in the AD brain,^43–45^ we recently showed that PNN loss is prevalent in the human AD and murine 5xFAD brain, a widely used transgenic AD mouse model,^46,47^ and correlates with plaque load.^11^ In line with this, de Vries et al. found that expression of ACAN is reduced in the frontal cortex of individuals with AD.^48^ Using colony-stimulating factor 1 receptor inhibitor treatment to investigate the role of microglia in AD, we showed that depletion of microglia prevents loss of PNNs and that plaque load is associated with reduced PNN density.^11^ These data suggest not only that microglia may be mediators of PNN loss in AD but also that PNNs may be protective against plaque deposition. Given this interplay of plaque formation, the microglial response, and ECM changes, we were interested in further exploring the role of structural and compositional ECM changes during AD.

To investigate how PNN deficits impact AD pathology, we generated 5xFAD^+/−^/*Acan*^fl/fl^/*Nestin*-Cre^+/−^ by crossing 5xFAD mice to floxed *Acan* and *Nestin*-Cre mice,^49^ which results in loss of *Acan* in neural lineage cells. Here, we show that 5xFAD^+/−^/*Acan*^fl/fl^/*Nestin*-Cre^+/−^ mice exhibit loss of ACAN, a critical component of PNNs, resulting in brain-wide PNN ablation. 5xFAD^+/−^/*Acan*^fl/fl^/*Nestin*-Cre^+/−^ mice exhibit enhanced plaque accumulation, impaired microglial association to plaques and an increased disease-associated microglia (DAM) phenotype. Interestingly, these mice also display reduced dystrophic neurites, suggesting that the microglial barrier surrounding plaques may influence neuritic damage and that these two features can be experimentally dissociated. These findings implicate an important role of PNNs, via ACAN deletion, in mediating the microglial response to plaques, with important therapeutic implications for ECM modulation in AD.

## RESULTS

### Constitutive brain-wide ablation of PNNs via ACAN deletion

**Figure 1.**
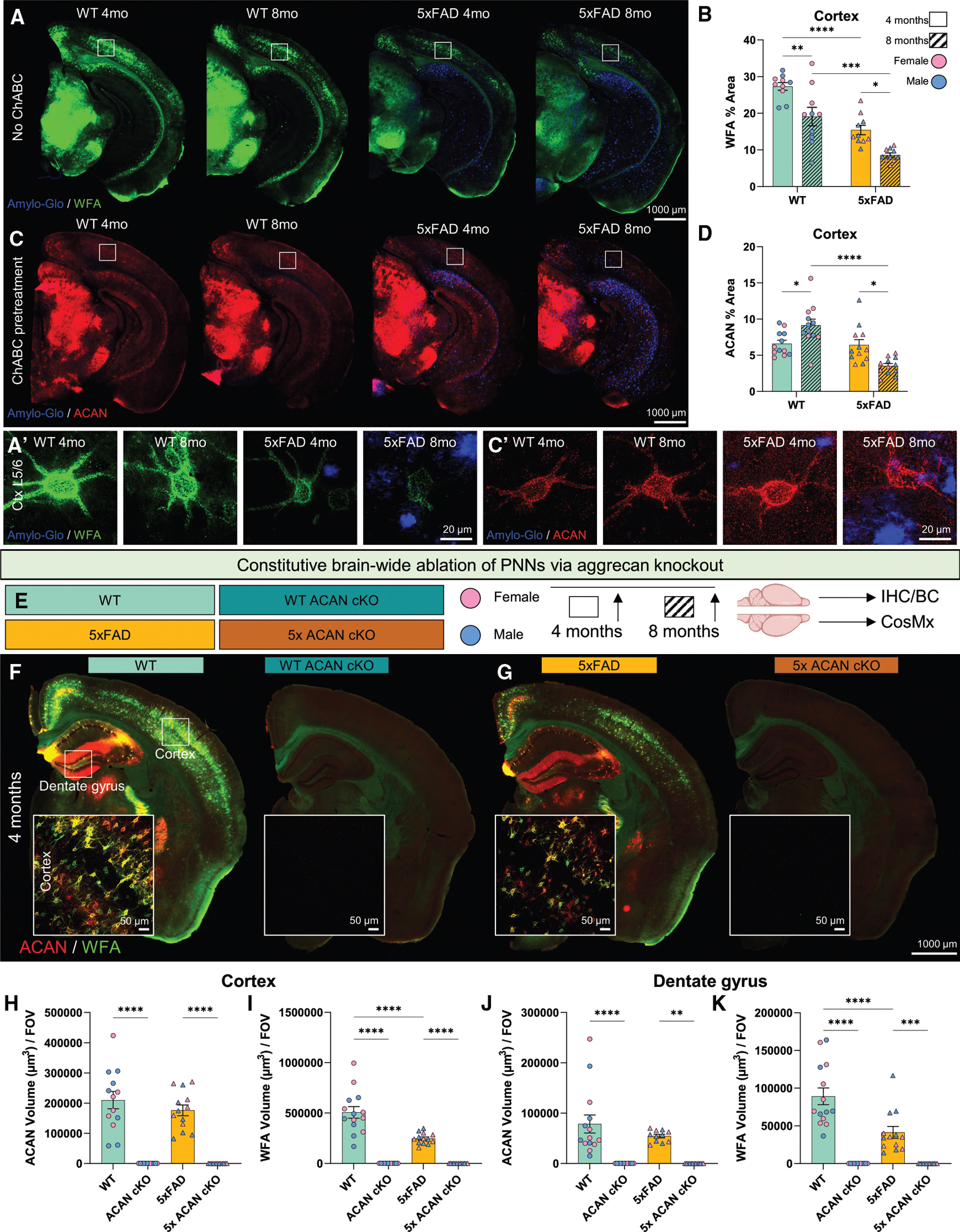
Constitutive brain-wide ablation of PNNs via *Acan* deletion (A and B) (A) Representative 5× magnification images of WT and 5xFAD murine hemibrains at 4 and 8 months stained for Amylo-Glo plaques and WFA^+^ PNNs (white boxes indicate FOVs selected) with (B) quantification of WFA % area for cortical FOVs. (C and D) (C) Representative 5× magnification images of WT and 5xFAD murine hemibrains at 4 and 8 months stained for Amylo-Glo plaques and ACAN^+^ PNNs following incubation of slices with chABC to expose ACAN core protein (white boxes indicate FOVs selected) with (D) quantification of ACAN % area for cortical FOVs. (A′ and C′) Super-resolution images of cortical layer 5/6 WFA^+^ PNNs (A′) and ACAN^+^ PNNs (C′). (E) Schematic of experimental groups and design. The four genotypes generated are *Acan*^fl/fl^ (WT), *Acan*^fl/fl^/*Nestin*-Cre^+/−^ (WT ACAN cKO), 5xFAD^+/−^/*Acan*^fl/fl^ (5xFAD), 5xFAD^+/−^/*Acan*^fl/fl^/*Nestin*-Cre^+/−^ (5x ACAN cKO). Brains were harvested at 4 and 8 months with *n* = 4–6 per sex per group; hemispheres were used for immunohistochemical (IHC), biochemical (BC), and spatial transcriptomic analyses. (F) Representative 5× magnification images with corresponding 20× cortical inset images of WT, WT ACAN cKO. (G) 5xFAD and 5x ACAN cKO brain sections stained for ACAN^+^ and WFA^+^ PNNs at 4 months. (H and I) (H) Quantification of cortical ACAN volume and (I) cortical WFA volume in all four genotypes at 4 months. (J and K) (J) Quantification of hippocampal ACAN volume and (K) hippocampal WFA volume in all four genotypes at 4 months. Statistical analysis used a two-way ANOVA with Tukey’s multiple comparisons correction for WFA and ACAN volume quantifications and a one-way ANOVA with Šídák’s multiple comparisons correction for ACAN and WFA volume in controls compared with knockouts. Significance indicated as *p < 0.05, **p < 0.01, ***p < 0.001. Data are represented as mean ± SEM. Scale bars, 1,000 μm (for whole brain images), 50 μm (for 20× confocal images), and 20 μm (for 63× super-resolution images).

We previously found that PNNs are extensively reduced in the 5xFAD mouse model, which develops amyloid pathology starting at age 1.5 months,^46^ as well as in human AD cortical tissue.^11^ To validate previous observations, we stained 4- and 8-month WT and 5xFAD brain tissue with Amylo-Glo to detect amyloid plaques, ACAN, which recognizes the core protein of the PNN CSPG ACAN, and WFA, which binds to GAG chains of CSPGs, to visualize PNNs. Here, we detect WFA^+^ PNNs throughout the murine brains with the highest densities of WFA^+^ PNNS in the midbrain/thalamus, retrospenial cortex, visual cortex, and subiculum (Figure 1A). To ensure accurate detection of ACAN, we next employed a chondroitinase ABC (chABC) pretreatment prior to immunostaining for ACAN to enzymatically remove GAG chains (Figures 1C, 1D, and S1). This approach allowed us to assess ACAN core protein levels without interference from GAG side chains. Here, we detect ACAN^+^ PNNs throughout the murine brain with the highest densities of PNNs in the midbrain/thalamus, retrosplenial cortex (RSctx), visual cortex, and subiculum (Figure 1C). We also provide super-resolution images of individual cortical layer 5/6 WFA^+^ PNNs (Figure 1A’) and ACAN^+^ PNNs (Figure 1C’). Assessment of cortical PNN coverage, measured by % area of WFA, highlights a significant reduction in 8-month WT and 5xFAD compared with their 4-month counterparts (Figures 1A and 1B). We also observe that 5xFAD exhibit reduced PNN coverage compared with age-matched WT controls (Figures 1A and 1B), indicating that aging and amyloid pathology impacts loss of PNNs or GAG chains on PNNs. To further assess this, we next quantified ACAN, measured by % area, and observed a significant increase in cortical ACAN^+^ PNN coverage in 8-month WT compared with 4-month WT (Figures 1C and 1D). Interestingly, in the presence of 5xFAD pathology, there is a significant decrease in cortical ACAN^+^ PNN coverage in 8-month 5xFAD compared with 4-month 5xFAD (Figures 1C and 1D). These findings show that under homeostatic conditions, increased age results in the loss of CS-GAG components, but not the loss of PNNs (i.e., the ACAN core of PNNs); while in the AD murine brain, CS-GAG components as well as core PNN protein loss is apparent. Together, these data validate previous findings and provide further characterization of PNN loss in the 5xFAD brain.

To understand how PNN loss impacts AD pathogenesis, we first generated *Acan*^fl/fl^ mice on the 5xFAD^+/−^ background. 5xFAD^+/−^/*Acan*^fl/fl^ mice were then crossed with *Nestin*-Cre^+/−^ mice as a genetic tool to delete *Acan*—the gene that encodes ACAN. Nestin is expressed by neural stem and progenitor cells,^50–52^ allowing us to restrict ACAN deletion to the brain and cells of neural lineage. Selective breeding of 5xFAD^+/−^/*Acan*^fl/fl^/*Nestin*-Cre^+/−^ mice to *Acan*^fl/fl^ mice resulted in the following four groups: *Acan*^fl/fl^ (WT), 5xFAD^+/−^/*Acan*^fl/fl^ (5xFAD), *Acan*^fl/fl^/*Nestin*-Cre^+/−^ (WT ACAN cKO), and 5xFAD^+/−^/*Acan*^fl/fl^/*Nestin*-Cre^+/−^ (5x ACAN cKO). All groups were sex-balanced with n = 4–6 mice per sex per group, total mouse body weight was recorded, and brains were harvested at age 4 and 8 months (Figures 1E and S2A). At 4 months, successful ACAN cKO was confirmed via ACAN and WFA labeling, revealing the absence of ACAN^+^ PNNs and WFA^+^ PNNs in WT ACAN cKO and 5x ACAN cKO mice (Figures 1F and 1G), as seen by the lack of ACAN^+^ and WFA^+^ PNN volume in the cortex and dentate gyrus (DG) (Figures 1H–1K). Of relevance, ACAN cKO also attenuates the formation of dandelion clock-like structures (DACS) detected by the CS-56 antibody,^53^ which are ECM structures distinct from PNNs (Figures S3A–S3D). Consistent with findings in cartilage matrix deficiency (*Cmd*^−/−^) mice, which have a mutation in the *Acan* gene,^54^ we found that both HAPLN1 and TNR are still broadly expressed in the non-aggregated ECM in WT ACAN cKO and 5x ACAN cKO (Figures S3E–S3H). Similarly, in line with previous work in the WT floxed *Acan* model^23^ we find that ACAN cKO prevents the aggregation of HAPLN1 and TNR to form PNN structures in the WT and 5xFAD background (Figures S3E–S3H, white boxed insets). These data show that ACAN cKO successfully ablates ACAN^+^ PNNs, WFA^+^ PNNs, as well as other structures of the ECM such as DACS, HAPLN1^+^ PNNs, and TNR^+^ PNNs.

### Absence of aggrecan leads to increased amyloid plaque load in 5xFAD mice

**Figure 2.**
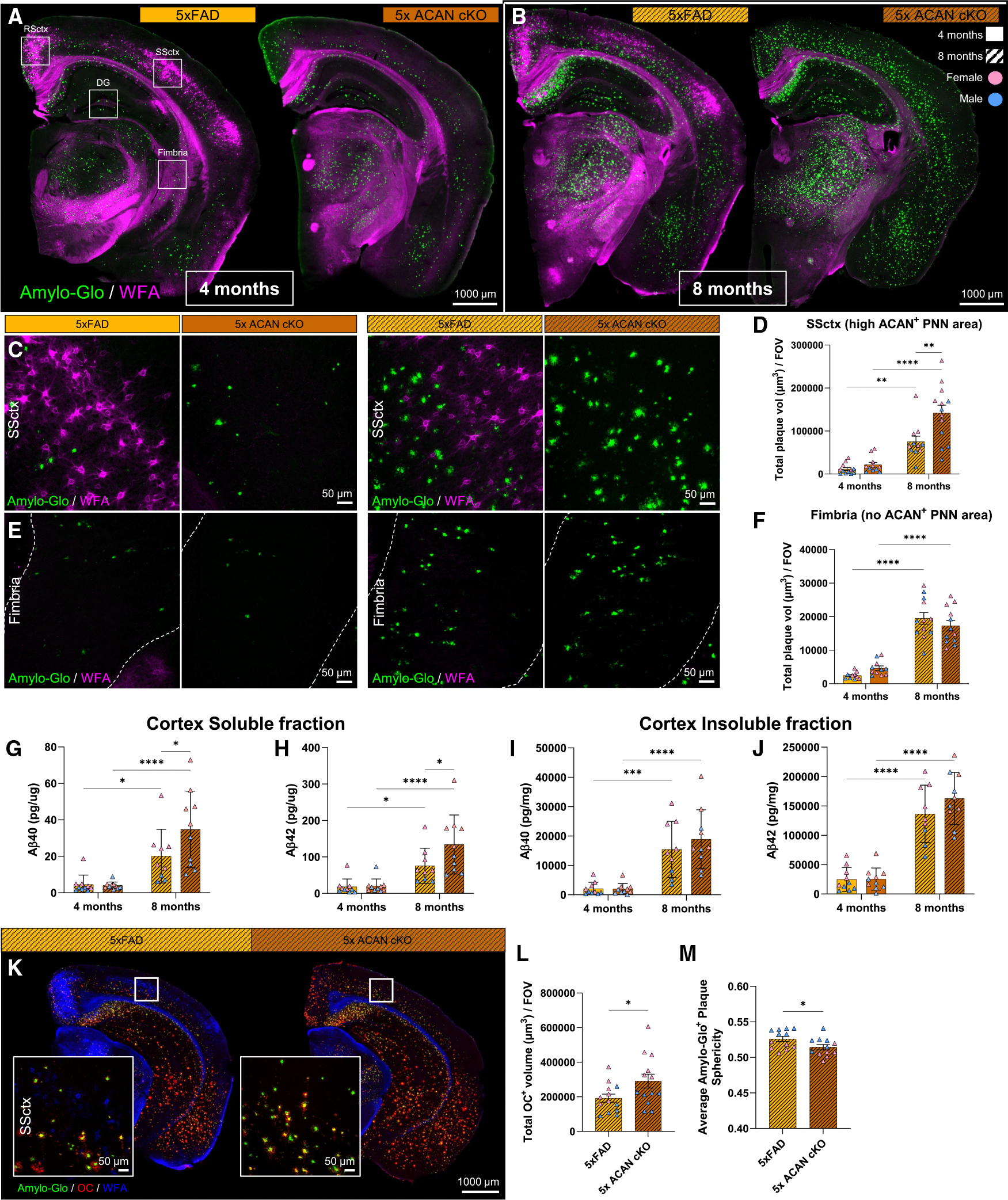
Absence of aggrecan leads to increased amyloid plaque load in 5xFAD mice (A and B) (A) Representative 5× magnification images of 5xFAD and 5x ACAN cKO brain sections stained for Amylo-Glo^+^ plaques and WFA^+^ PNNs at 4 months and (B) 8 months. White boxes indicate regions of interest for 20× confocal image analysis. (C and D) (C) Representative 20× confocal images of Amylo-Glo^+^ plaques and WFA^+^ PNNs in SSctx of 5xFAD and 5x ACAN cKO at 4 and 8 months with corresponding quantifications for (D) total plaque volume. (E and F) (F) Representative 20× confocal images of Amylo-Glo^+^ plaques and WFA^+^ PNNs in fimbria of 5xFAD and 5x ACAN cKO at 4 and 8 months with corresponding quantifications for (F) total plaque volume. (G) Quantification of cortical soluble Aβ40 shows a significant increase in concentration at 8 months in 5x ACAN cKO compared with 5xFAD. (H) Quantification of cortical soluble Aβ42 shows a significant increase in concentration at 8 months in 5x ACAN cKO compared with 5xFAD. (I) Quantification of cortical insoluble Aβ40. (J) Quantification of cortical insoluble Aβ42. (K) Representative 5× images of 5xFAD and 5x ACAN cKO stained for Amylo-Glo^+^ plaques, OC^+^ amyloid fibrils, and WFA^+^ PNNs at 8 months with corresponding 20× cortical inset images. (L and M) (L) Quantification of total OC^+^ volume per FOV and (M) average Amylo-Glo^+^ plaque sphericity per brain hemisphere. Statistical analysis used a two-way ANOVA with Tukey’s multiple comparisons correction for total plaque volume and cortical Aβ concentrations, and a two-tailed unpaired t test for OC volume and brain hemisphere quantifications. Significance indicated as *p < 0.05, **p < 0.01, ***p < 0.001. Data are represented as mean ± SEM. Scale bars, 1,000 μm (for whole brain images) and 50 μm (for 20× confocal images).

Using these generated transgenic mice, we next assessed how PNN loss, via ACAN cKO, impact the deposition and accumulation of Aβ plaques in 5xFAD mice, and stained 5xFAD and 5x ACAN cKO at 4 and 8 months with Amylo-Glo^55^ (Figures 2A and 2B). Specifically, we focused our initial analysis on the cortex, which is rich in PNNs and shows early Aβ deposition in the human brain and 5xFAD model.^56^ At 4 months, we found no significant differences in cortical Aβ plaque pathology, as measured by total somatosensory cortex (SSctx) plaque volume and number between the two groups (Figures 2C, 2D, and S4E). By age 8 months, there is a significant increase in total SSctx plaque volume and number in 5x ACAN cKO compared with 5xFAD (Figures 2C, 2D, and S4E). It is worth noting that, in the upper cortical layers, we observe fewer plaques in the presence of PNNs, and an increased plaque density in the absence of PNNs (Figure 2C). Collectively, these data reveal that loss of PNNs in a PNN-rich area of the brain exacerbates cortical Aβ pathology.

To further investigate the effects of ACAN loss in PNN non-rich areas of the brain, we expanded our analysis to include brain regions with varying levels of PNN density and ACAN expression. In PNN-rich regions where ACAN is highly expressed in PNN form, such as the RSctx, ACAN deletion also increased plaque load, which was more apparent in females (Figures S4A and S4B). In regions with minimal PNN deposition and diffuse ACAN expression, such as the DG, we observed a trending increase (*p* = 0.052) in plaque accumulation in 5x ACAN cKO compared with 5xFAD at 8 months (Figures S4C and S4D). In brain areas lacking both ACAN^+^ PNNs and diffuse ACAN, such as the fimbria, no differences in plaque burden were detected between 5xFAD and 5x ACAN cKO mice (Figures 2E, 2F, and S4F). These findings suggest that the impact of ACAN loss on Aβ pathology extends beyond highly organized PNNs and that both structured and diffuse ACAN could play a role in altered Aβ deposition.

To evaluate whether brain-wide deletion of *Acan* also affects soluble and insoluble Aβ levels, we further quantified Aβ levels using an ELISA Meso Scale Discovery (MSD) immunoassay in cortical brain homogenates collected from 4- and 8-month mice. In the cortical soluble fraction, we found a significant increase in soluble Aβ40 and Aβ42 levels in 8-month 5x ACAN cKO compared with 5xFAD (Figures 2G and 2H). However, no significant changes were found in insoluble Aβ40 and Aβ42 levels in 8-month 5x ACAN cKO compared with 5xFAD (Figures 2I and 2J). One consideration for the discrepancy between insoluble fractions and immunohistochemistry (IHC) is that the MSD ELISA assay measures total Aβ_40_ and Aβ_42_ levels from homogenized cortical lysates (including cortical regions that lack PNNs), whereas our IHC analyses focus on select brain regions enriched in PNNs.

To further investigate the impact of PNN loss, via ACAN cKO, on plaque composition/compaction, we used the conformation-specific OC antibody to visualize β sheet-rich amyloid fibrils surrounding dense core plaques. We stained 8-month 5xFAD and 5x ACAN cKO tissue with Amylo-Glo, OC, WFA, and observed a significant increase in total OC^+^ volume in 5x ACAN cKO compared with 5xFAD (Figures 2K and 2L), suggesting that the loss of PNNs, via ACAN cKO, promotes the accumulation of fibrillar Aβ as well as dense core plaques. Additionally, quantification of total plaques per brain hemisphere revealed a significant decrease in average plaque sphericity in 5x ACAN cKO compared with 5xFAD (Figure 2M), implicating changes in plaque compaction in the absence of ACAN and PNNs. Collectively, these data suggest that ACAN plays a critical role in Aβ plaque accumulation and compaction.

### Absence of aggrecan impairs the microglial response to plaques

**Figure 3.**
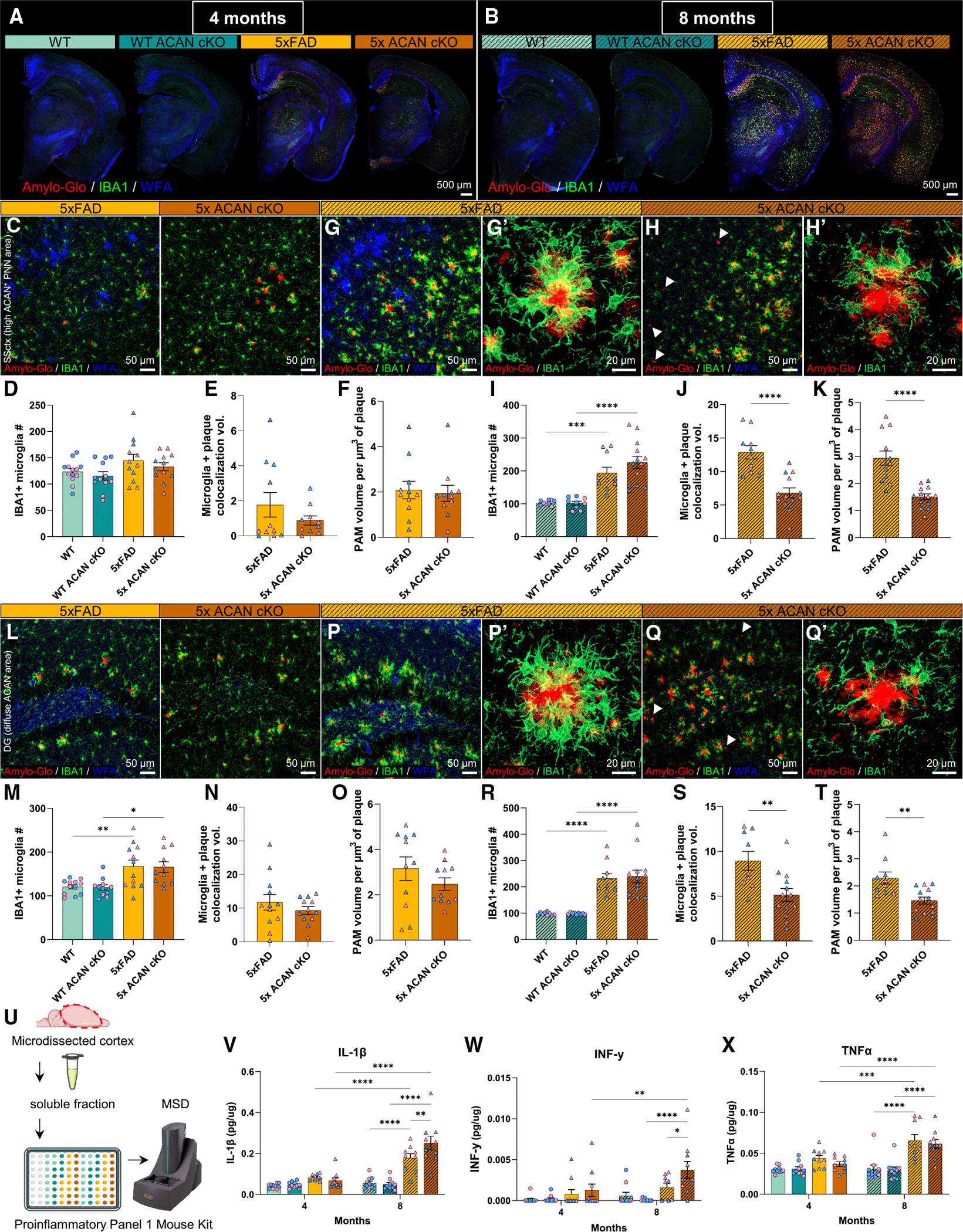
Absence of aggrecan impairs the microglial association with plaques (A and B) (A) Representative 5× magnification images of WT, WT ACAN cKO, 5xFAD, 5x ACAN cKO stained for Amylo-Glo^+^ plaques, IBA1^+^ microglia, and WFA^+^ PNNs at 4 months and (B) 8 months. (C) Representative 20× confocal images of plaques, microglia, and PNNs in 5xFAD and 5x ACAN cKO SSctx at 4 months. (D–F) (D) Quantification cortical microglia number in all four groups, (E) microglia and plaque colocalization volume in 5xFAD and 5x ACAN cKO, and (F) plaque-associated (PAM) volume per μm^3^ of plaque in 5xFAD and 5x ACAN cKO at 4 months. (G) Representative 20× confocal image of plaques, microglia, and PNNs in the 5xFAD cortex at 8 months. (G′) Representative 63× super-resolution image of plaques and microglia in the 5xFAD cortex at 8 months. (H) Representative 20× confocal image of plaques, microglia, and PNNs in the 5x ACAN cKO cortex at 8 months; white arrowheads highlight plaques lacking PAM. (H′) Representative 63× super-resolution image of plaques and microglia in the 5x ACAN cKO cortex at 8 months. (I–K) (I) Quantification of cortical microglia number in all four groups, (J) microglia and plaque colocalization volume in 5xFAD and 5x ACAN cKO, and (K) PAM volume per μm^3^ of plaque in 5xFAD and 5x ACAN cKO at 8 months. (L) Representative 20× confocal images of plaques, microglia, and PNNs in 5xFAD and 5x ACAN cKO DG at 4 months. (M–O) (M) Quantification of DG microglia number in all four groups, (N) microglia and plaque colocalization volume in 5xFAD and 5x ACAN cKO, and (O) PAM volume per μm^3^ of plaque in 5xFAD and 5x ACAN cKO at 4 months. (P) Representative 20× confocal image of plaques, microglia, and PNNs in the 5xFAD DG at 8 months. (P′) Representative 63× super-resolution image of plaques and microglia in the 5xFAD DG at 8 months. (Q) Representative 20× confocal image of plaques, microglia, and PNNs in the 5x ACAN cKO DG at 8 months; white arrowheads highlight plaques lacking PAM. (Q′) Representative 63× super-resolution image of plaques and microglia in the 5x ACAN cKO DG at 8 months. (R–T) (R) Quantification of DG microglia number in all four groups, (S) microglia and plaque colocalization volume in 5xFAD and 5x ACAN cKO, and (T) PAM volume per μm^3^ of plaque in 5xFAD and 5x ACAN cKO at 8 months. (U) Schematic illustrating the use of soluble fraction from microdissected cortices for cytokine analysis. (V) IL-1β concentration reveals a significant increase with age in the 5xFAD groups, a significant increase in the 5xFAD groups compared with the WT groups at 8 months, and a significant increase in 5x ACAN cKO compared with 5xFAD at 8 months. (W) INF-γ concentration reveals a significant increase with age for 5x ACAN cKO, a significant increase in 5x ACAN cKO compared with WT ACAN cKO at 8 months, and significant increase in 5x ACAN cKO compared with 5xFAD at 8 months. (X) TNF-α concentration reveals a significant increase with age for the 5xFAD groups, and significant increase in the 5xFAD groups compared with the WT groups at 8 months. Statistical analysis used a one-way ANOVA with Šídák’s multiple comparisons correction for microglia number, non-parametric Mann-Whitney test for 4-month microglia and plaque colocalization volume, and 4-month PAM volume per μm^3^ of plaque as data did not pass normality (Shapiro-Wilk test, *p* < 0.05), two-tailed unpaired t test for 8-month microglia and plaque colocalization volume, and PAM volume per μm^3^ of plaque. Significance indicated as *p < 0.05, **p < 0.01, ***p < 0.001. Data are represented as mean ± SEM. Scale bars, 500 μm (for whole brain images), 50 μm (for 20× confocal images), and 20 μm (for 63× super-resolution images).

Given the role of microglia in modulating plaque dynamics and indications of altered plaque compaction (i.e., altered plaque sphericity) in 5x ACAN cKO brains, we next used IHC to assess microglial changes following loss of ACAN. In this, we stained for Amylo-Glo^+^ plaques, IBA1^+^ microglia, and WFA^+^ PNNs in mice at age 4 and 8 months (Figures 3A and 3B). We first focused our analysis on the SSctx, where ACAN is present in the condensed PNN form. At 4 months, we observe no overt changes in cortical microglia in 5x ACAN cKO compared with 5xFAD (Figures 3C–3F and S4I). However, by 8 months, immunostaining for IBA1 reveals significant differences in cortical microglia (Figures 3G–3K and S4J), including increases in the number of IBA1^+^ cells and % area of microglia (i.e., IBA1 microglia coverage) in 5xFAD and 5x ACAN cKO compared with their respective WT controls (Figures 3I and S4J). Representative 20× images of the cortex in 5xFAD mice show microglia colocalize with plaques, highlighting their close physical association (Figure 3G). While we observe no differences in total microglia number and % area of microglia in 5xFAD vs. 5xFAD ACAN cKO mice (Figures 3I and S4J), we observe a significant reduction in direct contact between microglia and plaques as seen by a reduction in microglia and plaque colocalization volume (Figure 3J), suggesting an impaired microglia-plaque interaction. Interestingly, in the 5x ACAN cKO cortex at 8 months, we find multiple plaques lacking a microglial barrier (white arrowheads, Figure 3H). Microglia that surround amyloid plaques are known as plaque-associated microglia (PAM), and we find a significant decrease in PAM volume per μm^3^ of plaque in 5x ACAN cKO compared with 5xFAD (Figures 3G, 3H, and 3K), indicating a reduction in microglial clustering around plaques. Representative 63× super-resolution images of microglia and plaques in 5x ACAN cKO show both (1) less microglia surrounding a single plaque and (2) less microglia and plaque colocalization when compared with 5xFAD (Figures 3G′ and 3H′), indicating that ablation of PNNs, via ACAN cKO, leads to less PAM and potential impairment in microglia-plaque interactions. Next, we examined the DG, a region of the brain where ACAN is present in a non-aggregated diffuse form, allowing us to assess whether ACAN loss influences plaque accumulation beyond PNN-rich cortical areas. In DG microglia at 4 and 8 months, we observed a significant increase in the number of IBA1^+^ microglia in both 5xFAD groups compared with their respective WT controls, with no significant difference in the number or volume of microglia between PNN-intact controls and ACAN cKO groups (Figures 3L, 3M, 3P–3R, and S4K–S4L). These data confirm that ACAN deletion does not alter total microglia number nor IBA1^+^ microglia reactivity/morphology (Figures 3R and S4L). Similar to the cortex, in the 5xFAD DG at 8 months, we observe that microglia colocalize with plaques, highlighting their close physical association (Figure 3P), and, in the DG of 5x ACAN cKO brains, we find that the microglia-plaque interaction is also impaired, as seen by a significant reduction in microglia and plaque colocalization volume and PAM volume per μm^3^ of plaque (Figures 3Q, 3S, and 3T). These findings suggest that loss of ACAN (either in diffuse ACAN or ACAN^+^ PNN form) alters the ability of microglia to cluster around plaques.

To further probe how ACAN loss influences microglial function and activation, we performed cytokine profiling on the soluble fraction of 8-month cortical tissue using the MSD V-PLEX Proinflammatory Panel 1 Mouse Kit (Figure 3U). Of the cytokines measured, IL-1β (Figure 3V) and IFN-γ (Figure 3W) were significantly elevated in 5x ACAN cKO mice compared with 5xFAD, while other pro- and anti-inflammatory cytokines, including TNF-α (Figure 3X), showed no significant changes or were below detectable levels (Figures S5A–S5G). These results suggest that ACAN loss enhances specific proinflammatory responses in the plaque-rich cortex, which may contribute to impaired microglia-plaque interactions and increased plaque burden.

### Targeted spatial transcriptomics of the murine 5xFAD brain reveals microglia adopt an elevated DAM phenotype in the absence of aggrecan

**Figure 4.**
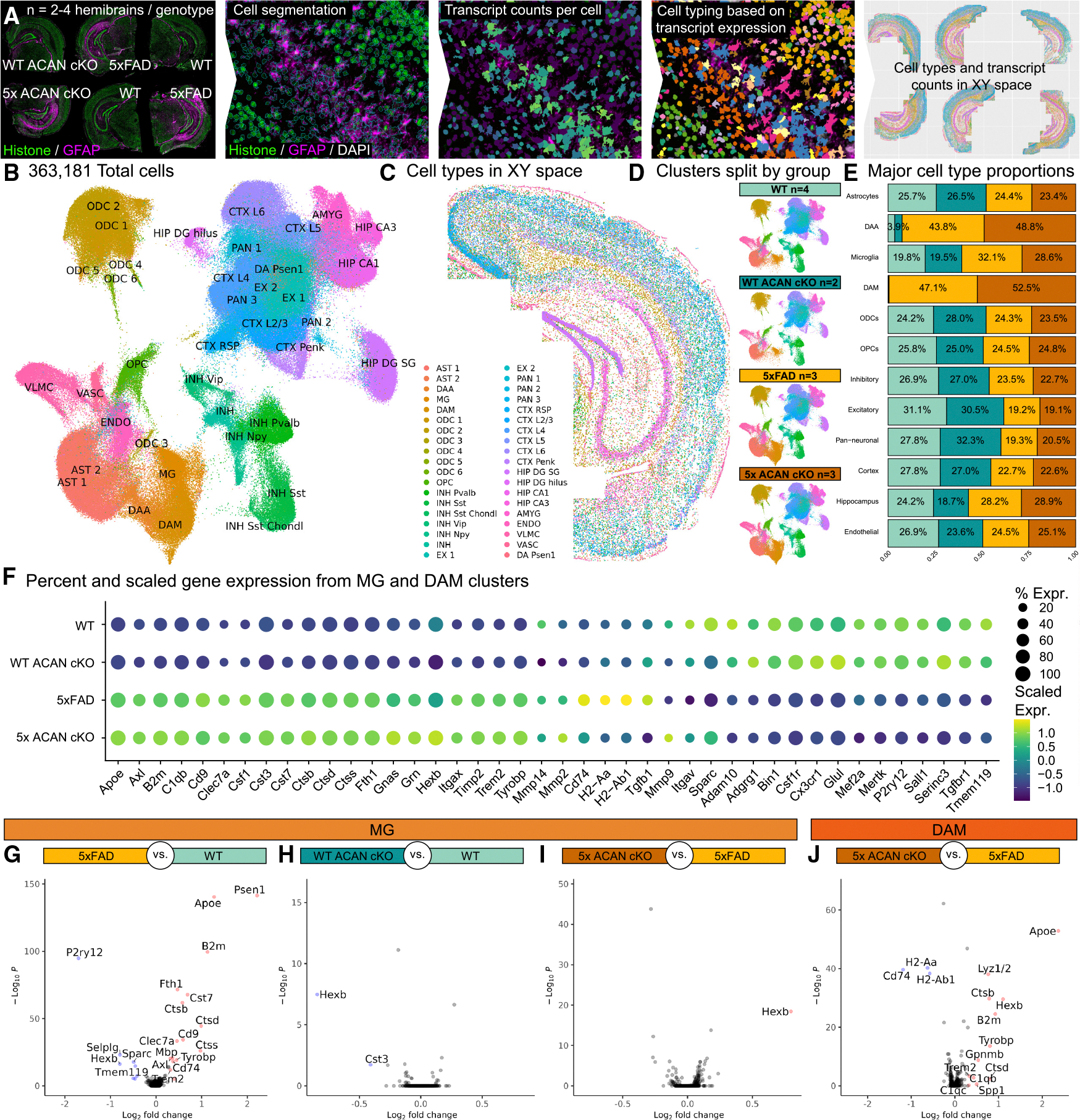
Targeted spatial transcriptomics of the murine 5xFAD brain reveals microglia adopt an elevated DAM phenotype in the absence of aggrecan (A) Workflow for targeted spatial transcriptomics via NanoString CosMx multiplexed 1000-plex mRNA Mouse Neuroscience panel. Slides with 10-μm-thick brain sections (six brains per slide, two slides) undergo tissue processing, cell segmentation, and data acquisition (detailed in the STAR Methods) resulting in transcript counts per cell which are used to classify cell type. Cell types and transcript counts can then be visualized in XY space. (B) Uniform manifold approximation and projection (UMAP) for dimension reduction of transcriptomic data from 363,181 cells results in 38 distinct clusters. (C) Thirty-eight clusters mapped in XY space on a WT brain. (D) UMAP containing all cells is split by group where WT *n* = 4, WT ACAN cKO *n* = 2, 5xFAD *n* = 3, and 5x ACAN cKO *n* = 3. DAA and DAM clusters are only present in 5xFAD background groups. (E) Stacked histogram displaying normalized proportions of major cell types (i.e., “Astrocytes” = AST 1 + AST 2). (F) Dot plot displaying percent and scaled expression of microglia-associated genes across genotypes in the MG and DAM clusters. (G–J) (G) Volcano plots for differentially expressed genes in 5xFAD vs. WT microglia, (H) WT ACAN cKO vs. WT microglia, (I) 5x ACAN cKO vs. 5xFAD microglia, and (J) 5x ACAN cKO vs. 5xFAD DAM.

To gain insight into how the absence of ACAN facilitates plaque accumulation and alters PAM, we next employed spatial transcriptomics. We conducted our study using the CosMx Spatial Molecular Imager, a multiplex *in situ* hybridization-based system that enables us to obtain gene expression data of 1,000 genes (Mouse Neuroscience Panel) at single-cell resolution. Here, we selected 8-month-old female brains for spatial transcriptomics as these mice exhibit the most pronounced plaque pathology, which is exacerbated with ACAN cKO, thus maximizing our ability to detect transcriptional changes in response to ACAN cKO in a high-plaque-load context. Coronal hemibrain slices were processed, capturing cortical and hippocampal brain regions of high PNN and plaque density, which resulted in a dataset encompassing 363,181 total cells from 12 brains. Cell segmentation was performed based on histone, ribosomal RNA, GFAP, and DAPI staining, and transcript counts were obtained with an average of 879 transcripts detected per cell (Figure S6). Linear dimensional reduction was performed using principal-component analysis. Next, cell clustering was performed using a community detection approach on a k-nearest neighbor graph, followed by non-linear dimensional reduction via uniform manifold approximation and projection (UMAP) for visualization. Clustering revealed 38 distinct cell populations, which were manually annotated based on transcript expression (Figures 4A, 4B, and S7A) and anatomical location in XY space (Figures 4B, 4C, S6A–S6E, and S7B). Here, we identified three astrocyte (AST 1–2, disease-associated astrocyte [DAA]) clusters, two microglial (MG, DAM) clusters, six oligodendrocyte (ODC 1–6) clusters, one oligodendrocyte precursor (OPC) cluster, six inhibitory neuron (INH Pvalb, INH Sst, INH Sst Chondl, INH Vip, INH Npy, INH) clusters, two excitatory neuron (EX 1–2) clusters, three pan-neuronal (PAN 1–3) clusters, six cortical neuron (CTX RSP, CTX L2/3, CTX L4, CTX L5, CTX L6, CTX Penk) clusters, four hippocampal neuron (HIP DG SG, HIP DG hilus, HIP CA1, HIP CA3) clusters, one amygdala cluster, one endothelial cell cluster, one vascular and leptomeningeal cell cluster, one vascular cell cluster, and one DA Psen1 cluster (i.e., a small cluster only present in 5xFAD and 5x ACAN cKO mice with high *Psen1* expression) (Figures 4B and 4C). Separating UMAPs by group confirmed the presence of DAM—which include PAMs^57^—and DAA clusters under AD conditions, as seen by their presence exclusively in the two 5xFAD groups (Figure 4D). To visualize any potential broad changes in cell numbers, individual clusters were categorized into major cell types. For example, clusters ODC1-ODC6 were placed in the ODCs major cell type category. Plotting the proportions of the number of cells in each major cell type normalized for the total number of cells in each group (i.e., to account for differences in sample size) shows, as expected, a higher proportion of microglia, DAM, and DAA present in 5xFAD groups compared with WT groups (Figure 4E). We observe that the proportions of inhibitory, excitatory, pan-neuronal, and cortex major cell types decreased in both 5xFAD and 5x ACAN cKO mice (Figure 4E). All other major cell types display no substantial changes in normalized cell proportions between controls and their respective ACAN cKO groups (Figure 4E). Plots of normalized proportions for all 38 clusters are shown in Figure S7C. Within the MG and DAM clusters, percent expression and scaled expression of microglial genes across all four groups confirms the upregulation of disease-associated genes and downregulation of homeostatic genes in 5xFAD groups (Figure 4F).

To further evaluate the impact of ACAN loss on different CNS cell types in the context of AD, we next performed differential gene expression analysis across the following three comparisons: 5xFAD vs. WT, WT ACAN cKO vs. WT, and 5x ACAN cKO vs. 5xFAD. Volcano plots show differentially expressed genes (defined as *p*_adj_ < 0.05 and absolute average difference > 0.3) within each CNS cell type between the different comparisons (5xFAD vs. WT, Figure S8; WT ACAN cKO vs. WT, Figure S9; 5x ACAN cKO vs. 5xFAD, Figure S10). Specifically, for RNA expression changes in the microglia (MG) cluster, in the 5xFAD vs. WT comparison, we observe an upregulation in several DAM genes (e.g., *Apoe*, *Cst7*, *Clec7a*, *Axl*, *Tyrobp*, *Trem2*) and downregulation of homeostatic microglial genes (e.g., *P2ry12*, *Hexb*, *Tmem119*) (Figure 4G). Interestingly, we find that the MG cluster, in WT ACAN cKO compared with WT, display a downregulation of *Hexb* and *Cst3* (Figure 4H). Previous studies have shown that these two genes are involved in ECM remodeling^58,59^ with cystatin C (encoded by *Cst3*), as a potent inhibitor of cysteine proteases (e.g., cathepsins) responsible for ECM degradation.^58^ The downregulation of genes involved in ECM degradation and preservation, respectively, imply altered ECM homeostasis in the absence of ACAN. In comparing the MG cluster between 5x ACAN cKO and 5xFAD, we detect the upregulation of a single gene (*Hexb*) (Figure 4I). *Hexb* encodes the β-subunit of β-hexosaminidase, a lysosomal enzyme that contributes to GAG chain catabolism^59^ and is recognized as a stably expressed core microglial gene.^60^ Supporting its role in lysosomal function, *Hexb* deficiency leads to disrupted lysosomal morphology in both microglia, highlighting its importance in maintaining lysosomal integrity.^61^ In our study, *Hexb* is upregulated in 5x ACAN cKO mice compared with 5xFAD, but downregulated in WT ACAN cKO mice compared with WT. This bidirectional regulation of *Hexb* in our data highlights a context-dependent response to ACAN deletion and PNN disruption that likely reflects differences in microglial state under healthy vs. disease conditions. In 5xFAD mice, ACAN cKO may amplify microglial lysosomal activity already triggered by pathology whereas in WT mice lacking pathology, ACAN cKO may reduce the need for lysosomal function or shift microglia to a less active, more surveillance-like state, leading to lower *Hexb* expression.

Importantly, we find that DAMs (DAM cluster) in 5x ACAN cKO compared with 5xFAD upregulate DAM genes (*Apoe*, *Trem2*, *Tyrobp*, *Spp1*, *Gpnmb*, etc.) and downregulate MHC class II pathway-associated genes (*CD74*, *H2-Aa*, *H2-Ab1*) (Figure 4J). Given that several of these genes are implicated in TREM2-mediated DAM responses,^62,63^ these data could indicate an altered microglial activation state in the absence of ACAN during AD. Additionally, a reduction in genes associated with MHC class II pathway in DAMs also suggests that these cells may exhibit altered antigen presentation abilities, such as a less effective immune response to Aβ.

### Subclustering of microglia reveals reduction in antigen-presenting cells in the absence of aggrecan

**Figure 5.**
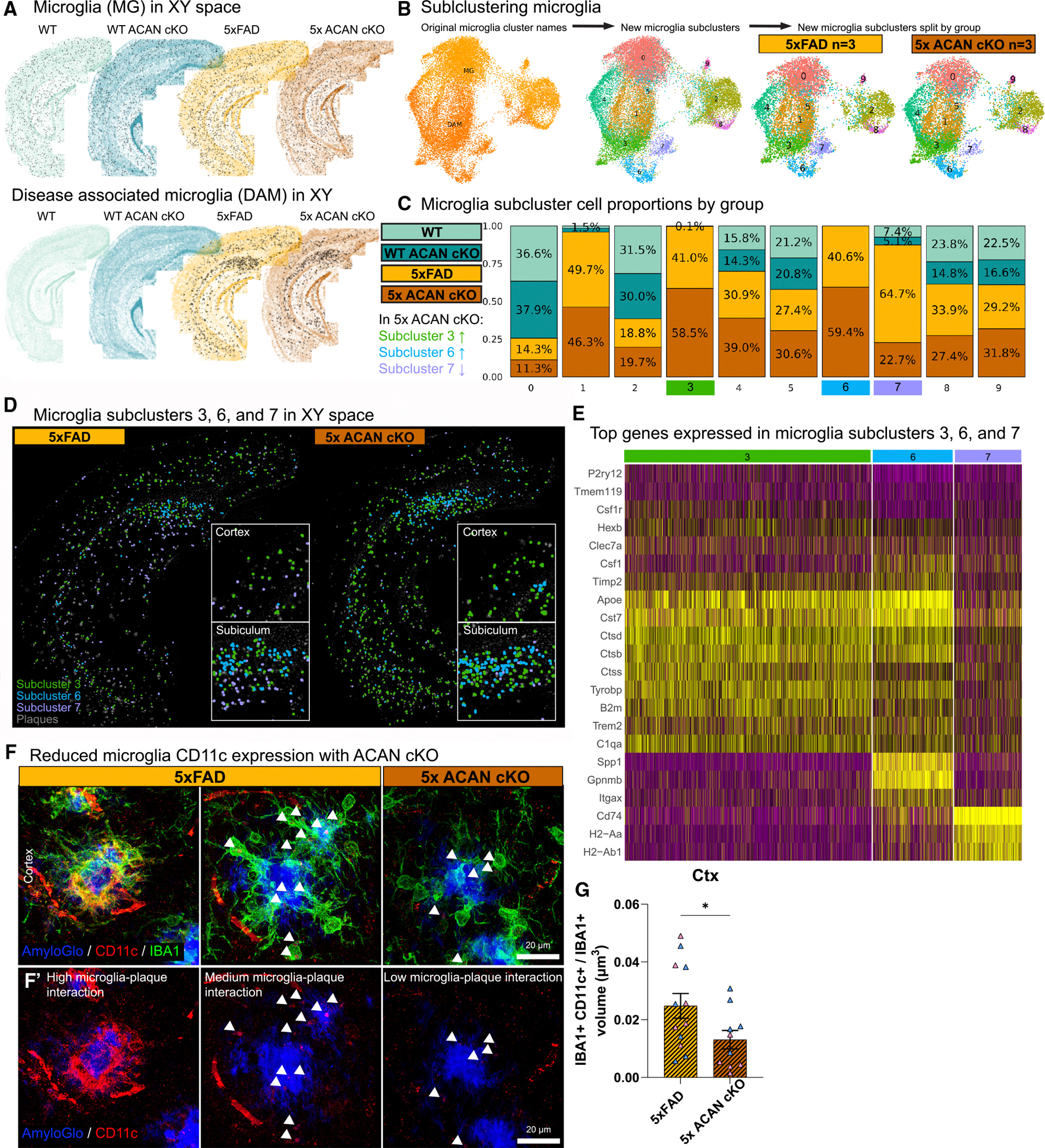
Subclustering of microglia reveals reduction in antigen presenting cells in the absence of aggrecan (A) The MG and DAM clusters plotted in XY space superimposed over the corresponding brain from each of the four groups, where each black point represents a cell. The MG cluster is present in all four groups, meanwhile the DAM cluster is only present in 5xFAD background groups. (B) UMAP demonstrating that a subset consisting of the original MG and DAM clusters were further clustered (i.e., subclustered) to produce microglia subclusters 0–9, which were then separated by group. (C) Stacked histogram displaying normalized proportions of microglia subclusters 0–9, with an emphasis on subclusters 3, 6, and 7. (D) Microglia subclusters 3 (green), 6 (light-blue), and 7 (light-purple) in XY space superimposed over an image of the corresponding brain with plaques labeled by DAPI. (E) Heatmap of microglial genes expressed in subclusters 3, 6, and 7. (F) Representative 63× super-resolution images of plaques (Amylo-Glo), CD11c, and microglia (IBA1) followed by (F′) plaques and CD11c only in 5xFAD and 5x ACAN cKO where white arrowheads highlight CD11c expression within microglia. (G) Quantification of IBA1^+^ CD11c^+^ volume normalized to total IBA1 volume per FOV. Statistical analysis used a two-tailed unpaired t test for IBA1 and CD11c volume quantifications. Significance indicated as *p < 0.05, **p < 0.01, ***p < 0.001. Data are represented as mean ± SEM. Scale bar, 20 μm (for 63× super-resolution images).

In the absence of ACAN, DAMs exhibit higher expression of DAM genes, but diminished ability to surround amyloid plaques as seen in 5x ACAN cKO compared with 5xFAD mice. To gain more insight into these microglial changes, and given that microglia are a heterogeneous population of cells,^64^ we next sought to explore whether this higher expression of DAM genes is observed across all DAMs or certain subpopulations of DAMs. To this end, we subclustered the original MG and DAM clusters from all 4 groups (Figure 5A), which resulted in 10 subclusters (Figure 5B). The cell number proportions revealed three subclusters of interest based on cell proportion changes above 20% between 5x ACAN cKO and 5xFAD: subclusters 3, 6, and 7 (Figure 5C). Specifically, in 5x ACAN cKO, we found a ~20% increase in microglia proportions in subclusters 3 and 6 and ~30% decrease in subcluster 7 compared with 5xFAD (Figure 5C). Moreover, we discovered that, spatially, DAM subclusters 3, 6, and 7 were all located within the vicinity of plaques, indicating their identity as PAMs (Figure 5D). Transcriptionally, DAM subclusters 3 and 6 are similar in that they exhibit a classic DAM signature (i.e., *Apoe*, *Cst7*, *Ctss*, *Tyrobp*, *Trem2*, etc.) with the exception of *Spp1*, *Gpnmb*, and *Itgax*, which are predominantly upregulated in subcluster 6 (Figure 5E). By contrast, DAM subcluster 7 is characterized by high expression of MHC class II pathway-associated genes (*CD74*, *H2-Aa*, and *H2-Ab1*) yet is reduced by almost two-thirds in 5x ACAN cKO (Figure 5E). To further assess whether DAMs or microglia surrounding plaques in 5x ACAN cKO exhibited altered antigen presentation, we next stained for CD11c, also known as integrin alpha X (ITGAX). CD11c is a cell surface receptor that has critical involvement in antigen presentation.^65–67^ Previous studies have shown that CD11c is upregulated in DAMs and typically found in close association with Aβ plaques.^68^ Staining for CD11c reveals high expression of CD11c in microglia surrounding plaques that are highly associated or interacting with Aβ plaques (i.e., forming a tight barrier around amyloid plaques; Figure 5F). Moreover, we observe a significant decrease in CD11c volume per microglia in 5x ACAN cKO compared with 5xFAD (Figure 5G), indicating that microglia in 5x ACAN cKO express less CD11c around amyloid plaques. While the DAM subcluster 6 cell proportion, characterized by elevated expression of *Spp1*, *Gpnmb*, and *Itgax*, is increased in 5x ACAN cKO mice compared with 5xFAD (Figures 5C–5E), via immunostaining for CD11c (encoded by *Itgax*) we find a reduction in IBA1^+^CD11c^+^/IBA1^+^ volume in 5x ACAN cKO mice relative to 5xFAD (Figures 5F and 5G). The discrepancy between *Itgax* expression in microglia subcluster 6 (Figure 5E) and lower CD11c^+^ immunostaining relative to total microglia (Figure 5G) likely reflects the restricted expression of *Itgax* in a specific microglial subset, whereas CD11c^+^ microglia, which label DAMs and PAMs, represent only a fraction (~20%) of the activated microglial pool.^62^ As such, reduced CD11c expression in 5x ACAN cKO mice pertains to the broader IBA1^+^ microglial population rather than subcluster-specific changes. Differences in protein detection, post-transcriptional regulation, or transient CD11c expression may also contribute to the divergence between transcriptomic and histological findings. Collectively, these data suggest that the absence of ACAN leads to a significant reduction in antigen presentation-related genes and cell surface marker expression by microglia, indicative of a compromised ability to engage in effective immune response.

### The absence of aggrecan alters dystrophic neurites in AD

**Figure 6.**
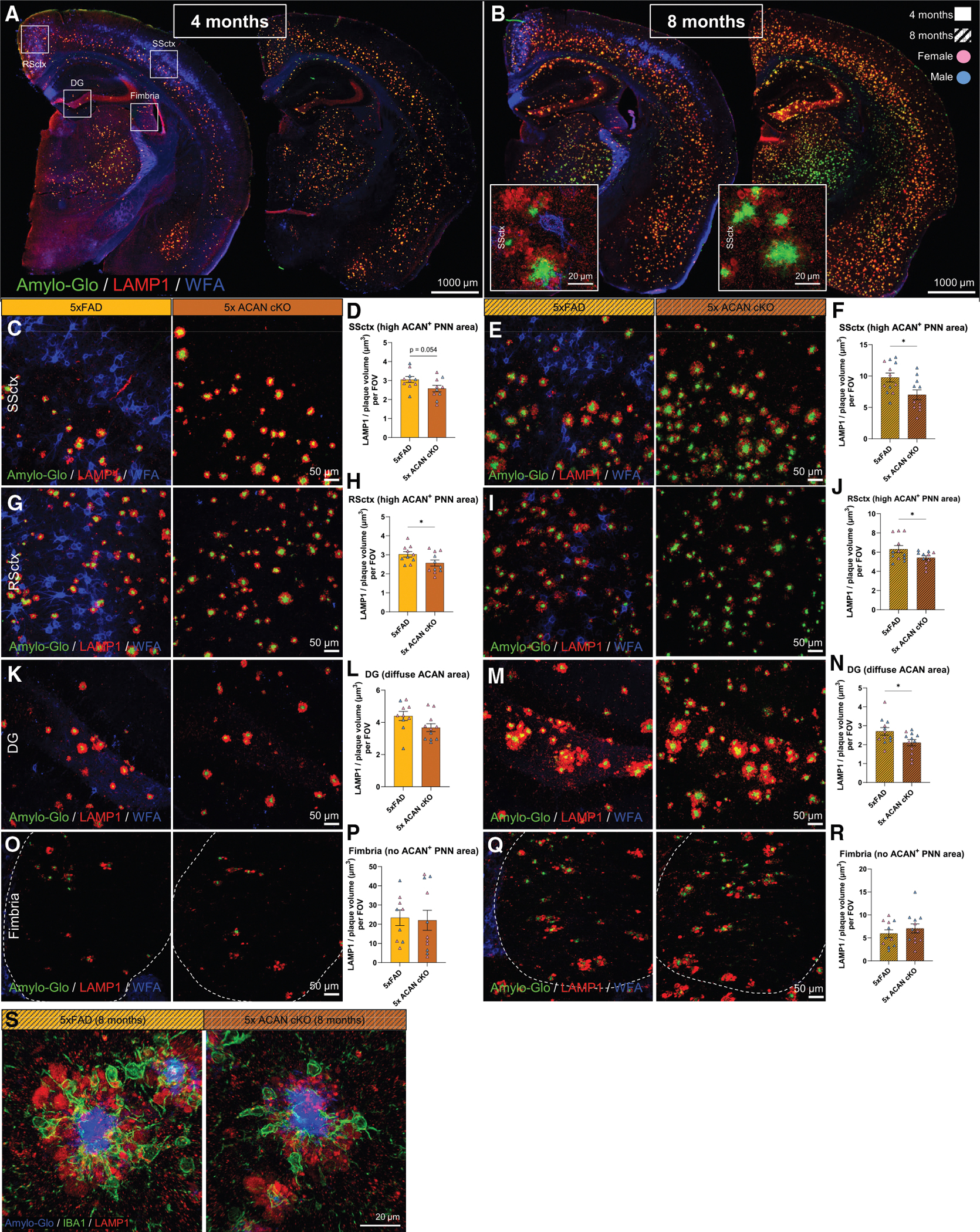
The absence of aggrecan alters dystrophic neurites in Alzheimer’s disease (A and B) (A) Representative 5× magnification images of 5xFAD and 5x ACAN cKO brain sections at 4 months and (B) 8 months stained for Amylo-Glo^+^ plaques, LAMP1^+^ dystrophic neurites, and WFA^+^ PNNs with cortical super-resolution images shown in white-boxed insets for 8-month groups. (C and D) (C) Representative 20× confocal images of SSctx Amylo-Glo^+^ plaques, LAMP1^+^ dystrophic neurites, and WFA^+^ PNNs from 4-month 5xFAD and 5x ACAN cKO mice with (D) quantification of LAMP1 volume normalized to plaque volume per FOV. (E and F) (E) Representative 20× confocal images of SSctx Amylo-Glo^+^ plaques, LAMP1^+^ dystrophic neurites, and WFA^+^ PNNs from 8-month 5xFAD and 5x ACAN cKO mice with (F) quantification of LAMP1 volume normalized to plaque volume per FOV. (G and H) (G) Representative 20× confocal images of RSctx Amylo-Glo^+^ plaques, LAMP1^+^ dystrophic neurites, and WFA^+^ PNNs from 4-month 5xFAD and 5x ACAN cKO mice with (H) quantification of LAMP1 volume normalized to plaque volume per FOV. (I and J) (I) Representative 20× confocal images of RSctx Amylo-Glo^+^ plaques, LAMP1^+^ dystrophic neurites, and WFA^+^ PNNs from 8-month 5xFAD and 5x ACAN cKO mice with (J) quantification of LAMP1 volume normalized to plaque volume per FOV. (K and L) (K) Representative 20× confocal images of DG Amylo-Glo^+^ plaques, LAMP1^+^ dystrophic neurites, and WFA^+^ PNNs from 4-month 5xFAD and 5x ACAN cKO mice with (L) quantification of LAMP1 volume normalized to plaque volume per FOV. (M and N) (M) Representative 20× confocal images of DG Amylo-Glo^+^ plaques, LAMP1^+^ dystrophic neurites, and WFA^+^ PNNs from 8-month 5xFAD and 5x ACAN cKO mice with (N) quantification of LAMP1 volume normalized to plaque volume per FOV. (O and P) (O) Representative 20× confocal images of fimbria Amylo-Glo^+^ plaques, LAMP1^+^ dystrophic neurites, and WFA^+^ PNNs from 4-month 5xFAD and 5x ACAN cKO mice with (P) quantification of LAMP1 volume normalized to plaque volume per FOV. (Q and R) (R) Representative 20× confocal images of fimbria Amylo-Glo^+^ plaques, LAMP1^+^ dystrophic neurites, and WFA^+^ PNNs from 8-month 5xFAD and 5x ACAN cKO mice with (R) quantification of LAMP1 volume normalized to plaque volume per FOV. (S) Representative 63× super-resolution images of Amylo-Glo^+^ plaques, IBA1^+^ microglia, and LAMP1^+^ dystrophic neurites in 5xFAD and 5x ACAN cKO at 8 months. Statistical analysis used a two-tailed unpaired t test for LAMP1 volume normalized to plaque volume quantifications. Significance indicated as *p < 0.05, **p < 0.01, ***p < 0.001. Data are represented as mean ± SEM. Scale bars, 1,000 μm (for whole brain images), 50 μm (for 20× confocal images), and 20 μm (for 63× super-resolution images).

Accumulating evidence has shown that alterations in the microglial barrier have an impact on plaque compaction and neuritic dystrophy.^69–72^ Therefore, we were next interested in determining how reductions in microglia surrounding plaques (that exhibit increased DAM gene expression) impact the development of dystrophic neurites in the absence of ACAN. Lysosomal-associated membrane protein 1 (LAMP1), a marker for dystrophic neurites, is a glycoprotein located in the lysosomal membrane of damaged neuronal processes found around plaques.^73^ Here, we stained for Amylo-Glo^+^ plaques, LAMP1^+^ dystrophic neurites, and WFA^+^ PNNs in 5xFAD and 5x ACAN cKO at both age 4 and 8 months (Figures 6A and 6B). At 4 months we observe no differences in LAMP1^+^ volume per μm^3^ of plaque in the SSctx (Figures 6C and 6D). However, by 8 months, we found LAMP1^+^ volume per μm^3^ of plaque is significantly decreased in SSctx (Figures 6E and 6F), suggesting less neuritic dystrophy in 5x ACAN cKO compared with 5xFAD mice. These findings suggest that reduced microglial coverage around plaques may also be associated with decreased neuronal damage, as indicated by fewer dystrophic neurites. Indeed, 63× super-resolution images of plaques and dystrophic neurites in the cortex at 8 months revealed a reduction of LAMP1^+^ signal surrounding plaques (Figure 6B, white boxed insets). Additionally, we found no significant differences in cortical thickness between 5xFAD and 5x ACAN cKO mice at either time point (Figure S2B), suggesting that ACAN loss does not result in cortical atrophy in the 5xFAD model. In the RSctx, where there is a high density of ACAN^+^ PNNs, we also observed significant reductions in LAMP1^+^ volume per μm^3^ of plaque in 5x ACAN cKO compared with 5xFAD at 4 and 8 months (Figures 6G–6J). In the DG, where ACAN exists in the diffuse ECM, we found LAMP1^+^ volume per μm^3^ of plaque in 5x ACAN cKO is only reduced at 8 months (Figures 6K–6N). However, in a region traditionally lacking diffuse ACAN and ACAN^+^ PNNs, such as the fimbria, we find no significant differences in LAMP1^+^ volume per μm^3^ of plaque between 5x ACAN cKO and 5xFAD at both ages (Figures 6O–6R). In staining for Amylo-Glo^+^ plaques, IBA1^+^ microglia, and LAMP1^+^ dystrophic neurites, 63× super-resolution images reveal an impaired microglial barrier and reduced dystrophic neurites surrounding plaques of similar size in the SSctx of 5x ACAN cKO vs. 5xFAD at 8 months (Figure 6S). Together, these findings provide evidence that the absence of ACAN hinders the microglial response to plaques, leading to altered dynamics in the formation of dystrophic neurites. These findings underscore the contribution of PAMs, or microglia that surround plaques, in exacerbating neuronal process damage, highlighting a consequence of disrupted microglial function in AD progression.

## DISCUSSION

In this study, we provide evidence that PNNs, via loss of ACAN, play a pivotal role in the microglial response to amyloid plaques in a mouse model of AD. PNNs are best known for regulating neural circuitry and restricting synaptic plasticity, with CSPGs and other PNN components also playing important roles in cell-matrix adhesion and migration.^12,74^ Recent years have highlighted PNN deficits in multiple neurological and neurodegenerative diseases,^35–40^ including AD.^11,44,45,48,75^ Previously, we reported that PNN staining is reduced in human AD and 5xFAD brain tissue, and that this reduction correlates with plaque load.^11^ Specifically, we showed that plaque deposition is inhibited in regions of high PNN density, indicative of a potentially protective effect of PNNs in AD. Beyond these studies, however, it remains unclear how the ECM causally influences the progression of AD, despite being the site where plaques form and the environment in which cell-to-cell signaling occurs. In our study, we sought to determine the consequences of PNN loss in AD and its effects on plaque pathology.

In this study, we confirmed previous findings that PNNs, as measured by WFA^+^ staining, are disrupted with aging and in 5xFAD mice. We also expanded these findings and showed deficits in ACAN^+^ PNNs in the 5xFAD model, suggesting that PNN loss in AD extends beyond CS-GAG compositional changes. By ablating PNNs via conditional knockout of the core PNN component ACAN from nestin^+^ and nestin-derived cells, we identified that the absence/disruption of PNNs leads to altered gene expression in the CNS, and increases extracellular plaque accumulation in AD, indicating PNNs may be protective against plaque pathology. As we observe significant increases in plaque burden in the 8-month 5x ACAN cKO mice, these findings suggest that disease progression-dependent mechanisms (i.e., additional factors or events occurring between 2 and 8 months) may influence the effects of ACAN loss on AD pathology. Given that plaques do not form in PNN-rich areas until after age 4 months, substantial changes would not be expected prior to that time point. Through spatial transcriptomics, we found that DAMs in 5x ACAN cKO mice upregulate genes associated with microglial activation (*Apoe*, *Lyz1/2*, *Ctsb*, *B2m*, *Tyrobp*, *Trem2*, etc.), while downregulating genes associated with antigen presentation (*H2-Aa*, *H2-Ab1*, *CD74*) compared with 5xFAD. Subclustering of DAMs revealed changes in cell number proportions, highlighting that the DAM population characterized by high expression of MHC class II pathway-associated genes is greatly reduced in the absence of ACAN. Consistent with this, we observed reductions in PAM and colocalization of microglia with plaques, suggesting the absence of ACAN in AD impairs the ability of microglia to surround plaques. Our findings suggest that PNN ablation, via ACAN loss, disrupts microglial function in AD, altering gene expression profiles linked to neuroinflammation and antigen presentation and ultimately exacerbating plaque accumulation. Altogether, this study provides evidence that ACAN regulates CNS gene expression beyond neurons and contributes to modulating the microglial response to extracellular amyloid plaques.

Here, we show that PNN ablation, via ACAN loss, has a detrimental effect on plaque pathology, but also observe that neuritic dystrophy is dampened. Contrary to these findings, studies involving enzymatic degradation of PNN components with chABC have shown neuroprotective effects, including restored LTP, increased synapse density near plaques, reduced amyloid burden, and improved memory performance.^76–78^ It has thus been proposed that PNN degradation in the aging or diseased brain may facilitate synaptic remodeling or plasticity, thereby serving a compensatory or neuroprotective function. However, it is important to note that PNNs regenerate within weeks after chABC treatment, limiting the ability to perform long-term studies required to evaluate disease progression such as plaque development in AD. Additionally, the resulting disaccharides from CSPG degradation with chABC give rise to a noncytotoxic activated microglial phenotype protective against experimental autoimmune encephalomyelitis, spinal cord injury, and neurotoxicity models^79–82^, which could explain reduced Aβ deposition at 5 months.^83^ Thus, these contrasting findings are likely due to the effects of acute PNN degradation vs. long-term ablation as explored in this study. Unlike chABC, which broadly degrades CSPGs and alters ECM composition, potentially triggering acute remodeling and microglial clearance, our model isolates ACAN loss without disturbing other matrix components. Additionally, while chABC is applied focally, our approach induces brain-wide ACAN depletion, enabling assessment of network-level effects. Moreover, converging evidence from genetic models and disease contexts suggests that sustained PNN loss and impairments often disrupt inhibitory synaptic architecture, increases neuronal excitability, and destabilizes network activity.^43,84,85^ PNN deficits in AD, epilepsy, and schizophrenia have been linked to reduced PV^+^ interneuron function and impaired circuit regulation.^43,86,87^ Together, these findings suggest that although PNN remodeling may be beneficial in specific contexts, chronic loss in neurodegenerative settings likely contributes to dysfunction rather than recovery.

In line with our study, investigations into the association between PNNs and tau pathology have found that neurons associated with ACAN^+^ PNNs are protected against neurofibrillary tangles, and that hyperphosphorylated tau is exclusively observed in neurons devoid of PNNs,^88,89^ indicating that PNNs are neuroprotective in AD. Given recent work that has implicated microglia in PNN degradation, indicating that PNN loss is a downstream consequence of microglial activation rather than a protective adaptation,^11,88,89^ we postulate that PNN loss may reflect a maladaptive response to chronic neuroinflammation or downstream effect of disease progression, rather than a neuroprotective or compensatory process.

Insight into the connection between PNN loss, neuroinflammation, and dampened neuritic dystrophy is captured in our microglia clustering data. Our observation that PAM volume is reduced in 5x ACAN cKO mice at 8 months suggests that ACAN or PNNs may play a role in mediating microglial clustering around plaques. Since PAMs have been implicated in dystrophic neurite formation and neuronal damage, altered microglial distribution could influence the extent or nature of neuritic pathology.^69,71,72^ Our current study employed a genetic model of constitutive ACAN deletion, thus future studies using temporally controlled approaches could more precisely define the timing and progression of these effects. These findings underscore the need to understand how ECM remodeling intersects with glial function and amyloid pathology across disease progression, and future studies incorporating cognitive assessments will be important to directly link PNN disruption to functional deficits in AD.

Previous studies have highlighted the important role of microglia and the microglial barrier on protecting against amyloid-associated neurodegeneration^71,90^; however, microglia could be playing differential roles at different disease stages. Despite our observation of fewer microglia around plaques in the absence of PNNs, those present are in a more activated state, as evidenced by the upregulation of DAM genes. In addition to heightened activation, the upregulation of DAM genes may signify that these PAMs are attempting to address plaque-related pathology, potentially in a maladaptive way. Thus, further understanding of the interactions between Aβ plaques, glial responses, and the ECM at various disease stages (and pathologies) is warranted and could be crucial to identifying potential new therapeutic avenues. Strategies aimed at modulating ECM components or preventing their pathological remodeling could help in mitigating the progression of AD, such as, targeting specific ECM molecules or enzymes involved in ECM degradation could preserve neuronal health and function. While no methodologies have been identified that restore PNNs, future exploration of these technologies could offer a novel therapeutic avenue for AD treatments. Collectively, these findings underscore the importance of ACAN in mediating the microglial responses to Aβ pathology and highlight the potential of targeting ECM components as a therapeutic strategy to mitigate neuroinflammation and plaque accumulation in AD.

### Limitations of the study

A limitation of our study is the use of nestin-Cre, which leads to ACAN deletion broadly across neural lineage cells, including neurons and glia, making it difficult to distinguish the role of cell-type-specific ACAN. While PNNs are primarily associated with inhibitory neurons,^16^ the potential contribution of glial-derived ACAN remains unknown. Future studies employing cell-type-specific Cre lines would be valuable in dissecting the distinct contributions of neuronal vs. glial ACAN in AD pathology. Further, this Cre line is constitutive, which results in early-life deletion of ACAN. Given the role of the ECM in neural circuit formation,^91^ we cannot exclude the possibility that developmental alterations contribute to the observed phenotypes. However, given that *Acan* is expressed relatively late in development with neurons being the predominant cellular sites of *Acan* expression^92^ and that PNNs primarily mature postnatally and continue to evolve into adulthood,^93,94^ our results likely reflect disease-driven effects. Future studies utilizing inducible Cre lines would be valuable in determining the specific impact of adult-onset ACAN deletion.

While genetically targeting *Acan* provides valuable insights into the role of PNNs in AD, it does not account for changes in CSPG-GAG chain glycosylation and sulfation patterns observed in AD.^45^ Therefore, future studies should focus on elucidating the mechanisms through which changes in CSPG glycosylation and GAG sulfation patterns influence neuronal health and disease progression. Here, in WT mice, we observed a reduction in WFA^+^ PNN labeling from age 4 to 8 months, despite increasing ACAN immunoreactivity, suggesting that PNNs may undergo structural or biochemical modifications, such as deglycosylation or altered sulfation patterns of CSPG GAG chains, which can affect WFA labeling.^95,96^ While these data suggest that PNNs are deglycosylated in the healthy aging process, further experiments with more aged mice are necessary.

## RESOURCE AVAILABILITY

### Lead contact

Further information and requests for resources and reagents should be directed to and will be fulfilled by the lead contact, Kim Green (kngreen@uci.edu).

### Materials availability

This study did not generate new unique reagents, cell lines, or mouse lines. The 5x ACAN cKO mouse line was generated through established breeding strategies using publicly available lines. Additional details are available from the lead contact upon request.

### Data and code availability

- Single-cell spatial transcriptomics dataset is available on the Dryad data repository (https://doi.org/10.5061/dryad.z612jm6pw). Dataset is provided as two separate RDS files split by flowcell, which include raw and corrected counts for the RNA data, along with comprehensive metadata. Metadata include mouse genotype, sample ID, cell type annotations, sex, and X-Y coordinates of each cell.
- No original code was developed in this study. Further information and details about the code used in this study are available from the lead contact upon request.
- Any additional information required to reanalyze the data reported in this paper is available from the lead contact upon reasonable request.

## STAR★METHODS

### EXPERIMENTAL MODEL AND STUDY PARTICIPANT DETAILS

All animal experiments performed in this study were approved by the UC Irvine Institutional Animal Care and Use Committee (IACUC) and comply with the Animal Research: Reporting of *in Vivo* Experiments (ARRIVE) guidelines. Hemizygous floxed Acan mice (B6.Cg-ACANtm1c(EUCOMM)Hmgu>/Jwfa) were generously provided by Dr. Suneel Apte (Cleveland Clinic) and Dr. Marianne Fyhn (University of Oslo) and bred to homozygosity. 5xFAD mice (B6.CgTg(APPSwFlLon, PSEN1*M146L*L286V)6799Vas/Mmjax, MMRRC Strain #034848-JAX) were maintained as hemizygous mice.^46^ Two Nestin-Cre homozygous females (B6.Cg-Tg(Nes-cre)1Kln/J, Strain #:003771) were obtained from JAX, bred to hemizygosity and maintained as hemizygous mice. The final pairing to produce 4-month and 8-month cohorts used in this study consisted of Acan^fl/fl^ females paired with 5xFAD^+/−^/Acan^fl/fl^/Nestin-Cre^+/−^ males. Littermates were housed in groups of 2–5 per cage in a 12:12 h light/dark cycle, fed *ad libitum*, and aged until the harvest dates. All mice were sacrificed via CO_2_ inhalation and perfused transcardially with ice-cold 1x PBS. Brains were extracted and dissected down the midline. Hemispheres were either fresh-frozen on dry ice for biochemical analysis, flash-frozen in isopentane for spatial transcriptomics, or drop-fixed in 4% paraformaldehyde for immunohistochemical (IHC) analysis. Fixed brains were cryopreserved in 1x PBS +0.05% NaN_3_ (sodium azide) + 30% sucrose at 4°C, frozen and sectioned at 40 μm on a Leica SM2000 R sliding microtome and stored in a 1x PBS +30% glycerol +30% ethylene glycol solution at −20°C for subsequent IHC analyses.

### METHOD DETAILS

#### Immunohistochemistry

Primary antibodies/stains used and dilutions are as follows: WFA (1:1000, B-1355, Vector Labs), ACAN (1:200, AB1031, Millipore), OC (1:1000; AB2286; Sigma-Aldrich), IBA1 (1:1000, 019–19741, Wako; and 1:500, 234 009, Synaptic Systems), LAMP1 (1:200; AB25245, Abcam), CD11c (1:100.; 50–112–2633; eBioscience), CS-56 (1:200; ab11570, Abcam), CS-6 mAb clone 3B3 (1:20, PRPG-BC-M04, Cosmo Bio USA), HAPLN1 (1:50; AF2608, R&D Systems, Minneapolis, MN), TNR (1:200; 217 008, Synaptic Systems, Germany).

Amylo-Glo staining (TR-300-AG; Biosensis, Thebarton, South Australia, AU) was performed before blocking tissue and according to manufacturer’s instructions to visualize fibrillar Aβ plaques. Amylo-Glo is diluted from 100X stock to 1X in 0.9% saline solution. Brain sections were first immersed in 70% ethanol for 5 min at room temperature with rocking. Following this, the sections were washed in distilled water for 2 min without rocking. Subsequently, the sections were incubated in 1X Amylo-Glo staining solution for 10 min with rocking. The sections were then rinsed in 0.9% saline solution for 5 min without rocking and finally washed in distilled water for 15 s. Following these washes, the sections proceeded with the standard IHC protocol described below.

Additionally, for ACAN staining, tissue underwent antigen retrieval followed by a 2 day pretreatment incubation with chABC (C3667, Millipore Sigma) to enzymatically cleave CS-GAG chains and expose ACAN core protein: Tissue sections were rehydrated and antigen retrieval was performed by heating the sections in citrate buffer (10 mM; pH 6.0) for 30 min at 80°C followed by a 10-min cooling period. Sections were then incubated in freshly prepared chABC (250 mU/mL in 50 mM Tris-HCl, pH 8.0, with 0.1% BSA) at 37°C for 5 h, followed by a fresh change in chABC and incubation overnight at 4°C with gentle shaking. After digestion, tissues were washed in PBS before proceeding with the standard IHC protocols described below.

For HAPLN1 and TNR staining, antigen retrieval was performed using 1X Tris-EDTA buffer (pH 9.0; ab93684, Abcam, Cambridge, MA) at 95°C for 30 min before proceeding with the standard IHC protocols described below.

As previously described,^97^ sections were washed 3 × 5 min in 1x PBS and immersed in normal serum blocking solution (5% normal serum +0.2% Triton X-100 in 1x PBS) for 1 h. Tissue was then incubated overnight in primary antibody at the dilutions described above in normal serum blocking solution at 4°C. The next day tissue sections were washed in 3 × 5 min in 1x PBS before being placed in appropriate secondary antibody in normal serum blocking solution (1:200 for all species and wavelengths; Invitrogen) for 1 h. Tissue sections were then washed for 3 × 5 min in 1x PBS before tissue was mounted and cover slipped.

To capture whole brain stitches, automated slide scanning was performed using a Zeiss AxioScan.Z1 equipped with a Colibri camera and Zen AxioScan 2.3 software. High resolution fluorescent 20x images were obtained using a Leica TCS SPE-II confocal microscope and LAS-X software. Super resolution 63x fluorescent images were obtained using a Zeiss LSM 900 microscope equipped with Airyscan 2. Super resolution image acquisition and processing were performed using Zeiss ZEN Blue software. For image quantification, 3D reconstructions of the images were generated and analyzed using Imaris software (9.7.2). Key parameters such as volume and fluorescence intensity mean were quantified through automated segmentation and object tracking within the software. Additionally, colocalization analyses were performed to assess the spatial relationships between different markers. ImageJ software was also used to measure the percentage of area covered by fluorescent signals and to calculate the integrated density for specific regions of interest. The % area was determined by thresholding the images to isolate the fluorescent signal, followed by measuring the ratio of signal area to the total area. Integrated density, representing total signal intensity, was calculated by multiplying the mean gray value by the area of the selected regions.

#### Aβ soluble and insoluble fractions and neurofilament light-chain concentrations

As previously described,^47^ cortices from fresh-frozen hemispheres of minimum 4–6 females and males per age and per genotype were microdissected and pulverized using a Bessman Tissue Pulverizer. Pulverized cortex was homogenized in 1000 μL/150 mg of Tissue Protein Extraction Reagent (TPER; Life Technologies, Grand Island, NY). Protease and phosphatase inhibitors were added to the homogenized samples which were then centrifuged at 100,000 g for 1 h at 4°C to generate TPER-soluble fractions. For formic acid-fractions, pellets from TPER-soluble fractions were homogenized in 70% formic acid: half of used TPER volume for cortex. Following this, samples were centrifuged again at 100,000 g for 1 h at 4°C. Protein in the insoluble fraction of microdissected cortical tissue was normalized to its respective brain region weight, while protein in soluble fractions were normalized to the protein concentration determined via Bradford Protein Assay. Formic acid neutralization buffer was used to adjust pH prior to running ELISAs.

Quantitative biochemical analyses of human Aβ soluble and insoluble fraction levels were acquired using the V-PLEX Aβ Peptide Panel 1 (6E10) (K15200G-1; Meso Scale Discovery, Rockville, MD). Quantitative biochemical analysis of neurofilament-light chain (NfL) in plasma was performed using the R-Plex Human Neurofilament L Assay (K1517XR-2; Meso Scale Discovery). Quantitative biochemical analysis of cytokine levels in cortical soluble fraction was measured by the V-PLEX proinflammatory Panel 1 (mouse) Kits (K15048D; Meso Scale Discovery).

#### Single-cell spatial transcriptomics

Brain hemispheres harvested for spatial transcriptomics were placed on a pre-frozen flat spatula to maintain shape and then submerged in isopentane maintained at −40°C for a minute: 30 s on the spatula and the remaining 30 s off the spatula. The flash fresh-frozen brains were wrapped in aluminum foil and placed in pre-frozen airtight 15 mL conical tubes before being stored at −80°C. For sectioning, brain hemispheres were embedded in optimal cutting temperature (OCT) compound, and 10 μm thick coronal sections were prepared using a cryostat (Leica CM1950). Twelve hemibrains were mounted directly onto two VWR Superfrost Plus microscope slides (Avantor, 48311–703), 6 hemibrains per slide, and kept at −80°C until fixation. All mice used for the spatial transcriptomics experiment were 8-month females (WT *n* = 4, WT ACAN cKO *n* = 2, 5xFAD *n* = 3, 5x ACAN cKO *n* = 3). The samples were processed according to the Nanostring CosMx fresh-frozen slide preparation manual for RNA and protein assays.

#### Slide preparation for spatial transcriptomics

Slides were immersed in 10% neutral buffered formalin (NBF; CAT#15740) for 2 h at 4°C, washed three times in 1X PBS (pH 7.4) for 2 min each, then baked at 60°C for 30 min. Slides were processed as follows: three washes of 1X PBS for 5 min each, 4% sodium dodecyl sulfate (SDS; CAT#AM9822) for 2 min, three washes of 1X PBS for 5 min each, 50% ethanol for 5 min, 70% ethanol for 5 min, and two washes of 100% ethanol for 5 min each, before air drying for 10 min at room temperature. Antigen retrieval was performed in a pressure cooker at 100°C for 15 min in 1X CosMx Target Retrieval Solution (Nanostring, Seattle, WA). Slides were transferred to DEPC-treated water (CAT#AM9922) and washed for 15 s, incubated in 100% ethanol for 3 min, then air-dried for 30 min. Each slide was incubated with digestion buffer (3 μg/mL Proteinase K in 1X PBS; Nanostring) for tissue permeabilization, then washed twice in 1X PBS for 5 min each. Fiducials for image alignment were diluted to 0.00015% in 2X SSC-T and applied to the slide, then incubated for 5 min. Tissues were then post-fixed with the following washes: 10% NBF for 1 min, two washes of NBF Stop Buffer (0.1M Tris-Glycine Buffer, CAT#15740) for 5 min each, and 1x PBS for 5 min. Next, NHS-Acetate (100 mM; CAT#26777) mixture was applied to each slide and incubated for 15 min at RT. Slides were washed twice in 2X SSC for 5 min each. Slides were incubated with a modified 1000-plex Mouse Neuro RNA panel (Nanostring) for *in situ* hybridization along with an rRNA segmentation marker in a hybridization oven at 37°C for 16–18 h overnight. Following overnight *in situ* hybridization, slides were washed twice in a stringent wash solution (50% deionized formamide [CAT#AM9342], 2X saline sodium citrate [SSC; CAT#AM9763]) at 37°C for 25 min each, then twice in 2X SSC for 2 min each. Slides were incubated in DAPI nuclear stain for 15 min, washed in 1X PBS for 5 min, incubated with GFAP and histone cell segmentation markers for 1 h, then washed three times in 1X PBS for 5 min each. Flow cells were affixed to each slide to create a fluidic channel for imaging, then loaded into the CosMx instrument. Approximately 300 FOVs were selected per slide, capturing hippocampal and cortical regions for each hemibrain section. Slides were imaged for 7 days and data were uploaded to the Nanostring AtoMx platform. Proper cell segmentation was confirmed (Figures S3A–S3E). Pre-processed data was exported as a Seurat object (v5.0.1)^98^ for further analysis in R 4.3.1.

#### Spatial transcriptomics data analysis

Spatial transcriptomics data were filtered using the AtoMx RNA Quality Control module to flag outlier negative probes (control probes targeting non-existent sequences to quantify non-specific hybridization), lowly-expressing cells, FOVs, and target genes. Data were then normalized and scaled using Seurat SCTransform to account for differences in library size across cell types.^99,100^ Principal component analysis (PCA) and uniform manifold approximation and projection (UMAP) analysis were performed to reduce dimensionality and visualize clusters in space. Unsupervised clustering using a shared nearest neighbor clustering algorithm at 1.0 resolution yielded 38 clusters. Clusters were manually annotated based on gene expression and spatial location. Cell proportion plots were generated by first plotting the number of cells in each major cell type and scaling to 1. Normalized percentages for each genotype were calculated by dividing the number of cells in a given cell type-genotype pair by the total number of cells in that genotype, then dividing by the sum of the proportions across the cell type, to account for differences in genotype sample sizes (i.e., *n* = 3 for 5xFAD groups, but *n* = 4 for WT and *n* = 2 for WT ACAN cKO). Differential gene expression analysis per cell type between genotypes was performed on scaled expression data using MAST to calculate the average difference, defined as the difference in log-scaled average expression between the two groups for each major cell type.^101^ Microglia were subset and further subclustered for further analysis. Data visualizations were generated using ggplot2.^102^

### QUANTIFICATION AND STATISTICAL ANALYSIS

For IHC analyses, n = 4–6 mice per sex per genotype were included at both 4- and 8-month timepoints. For each stain, one coronal brain section per mouse was used. Within each section, one field of view (FOV) per brain region was analyzed, with up to four regions (e.g., cortex, hippocampus, etc.) assessed per section. For whole brain images separated by fluorescent channel for all main figures, see Figure S12. Quantification of each marker was completed in batch using IMARIS software (v9.7.2), serving as a form of blinding, as files were anonymized and processed uniformly without knowledge of group identity.

Statistical analyses were performed using GraphPad Prism (v9.0.0). Normality was assessed with the Shapiro-Wilk test prior to statistical comparison. When comparing two groups, unpaired two-tailed Student’s t-tests were used for normally distributed data, and Mann–Whitney U tests were used for non-parametric data. For multiple group comparisons, one-way ANOVA was used to assess differences between genotypes at a single age, while two-way ANOVA was employed to evaluate the effects of genotype and age (4 and 8 months) and their interaction. 4-month and 8-month tissue sections were stained and processed in separate batches for LAMP1 data and thus are not directly comparable in a unified two-way ANOVAs analysis. Post hoc analyses were performed using Tukey’s or Šidák’s test, as appropriate. Statistical significance was defined as *p* < 0.05. All data are presented as mean ± SEM, with significance denoted as follows: *p < 0.05, **p < 0.01, ***p < 0.001. Statistical trends were considered at *p* < 0.10, indicated with #.

## Supplementary Material

1

2

SUPPLEMENTAL INFORMATION

Supplemental information can be found online at https://doi.org/10.1016/j.celrep.2025.116064.

## Figures and Tables

**KEY RESOURCES TABLE:** 

REAGENT or RESOURCE	SOURCE	IDENTIFIER

Antibodies		

Biotinylated Wisteria Floribunda Lectin (WFA, WFL)	Vector Labs	Cat# B-1355; RRID:AB_2336874
rabbit-*anti*-ACAN	Millipore	Cat# AB1031; RRID:AB_90460
Amylo-Glo	Biosensis	Cat# TR-300-AG
rabbit-*anti*-Aβ fibrils (OC)	Sigma-Aldrich	Cat# AB2286; RRID:AB_1977024
rabbit-*anti*-IBA1	FUJIFILM Wako Pure Chemical Corporation	Cat# 019–19741; RRID:AB_839504
chicken-*anti*-IBA1	Synaptic Systems	Cat# 234 009; RRID:AB_2891282
rat-*anti*-LAMP1	Abcam	Cat# AB25245; RRID:AB_449893
Armenian hamster-*anti*-CD11c	eBioscience	Cat# 50–112-2633; RRID:AB_467115
mouse-*anti*-chondroitin sulfate (CS-56)	Abcam	Cat# AB11570; RRID:AB_298176
mouse-*anti*-6-sulfated unsaturated disaccharide neoepitopes of CS (Clone 3B3)	Cosmo Bio USA	Cat# PRPG-BC-M04
goat-*anti*-HAPLN1	R&D Systems	Cat# AF2608; RRID:AB_2116135
rabbit-*anti*-TNR	Synaptic Systems	Cat# 217 008; RRID:AB_3083013

Chemicals, peptides, and recombinant proteins		

Corn Oil	Sigma-Aldrich	Cat# C8267-500ML
Formaldehyde solution 4%, buffered, pH 6.9	Sigma-Aldrich	Cat# 1.00496.0700
Formalin, Neutral, Buffered 10% w/v in Phosphate Buffer	EMS Diasum	Cat# 15740
PBS - Phosphate-Buffered Saline (10X) pH 7.4	ThermoFisher	Cat# AM9625
TBS with Tween^™^ (TBST), 20X Solution	ThermoFisher	Cat# J77500.K2
10 mM Tris Base	FisherScientific	Cat# BP2471-500
1 mM EDTA	FisherScientific	Cat# S311-500
Tween 20	FisherScientific	Cat# BP337-500
4% sodium dodecyl sulfate	ThermoFisher	Cat# AM9822
DEPC-treated water	ThermoFisher	Cat# AM9922
50% deionized formamide	ThermoFisher	Cat# AM9342
2X saline sodium citrate	ThermoFisher	Cat# AM9763
Fluoromount-G	Southern Biotech	Cat# 0100-01
Fluoromount-G with DAPI	Invitrogen	Cat# 00-4959-52
Chondroitinase ABC (chABC)	Millipore Sigma	Cat# C3667

Critical commercial assays		

V-PLEX Aβ Peptide Panel 1 (6E10) Kit	Meso Scale Discovery	Cat# K15200G-1
R-Plex Human Neurofilament L Assay	Meso Scale Discovery	Cat# K1517XR-2
V-PLEX Proinflammatory Panel 1 Mouse Kit	Meso Scale Discovery	Cat #K15048D

Deposited data		

Single-cell spatial transcriptomics of ACAN cKO in WT and 5xFAD mice	Dryad	https://doi.org/10.5061/dryad.z612jm6pw

Experimental models: Organisms/strains		

Mouse: *C57BL/6 (Wild-fype)*		JAX 000664
Mouse: B6.Cg-Tg(APPSwFlLon, PSEN1*M146L*L286V) 6799Vas/Mmjax (5xFAD)	Oakley et al.^46^	JAX 034848
Mouse: B6.Cg-ACANtm1c(EUCOMM)	Rowlands et al.^23^	MGI: 6160877
Hmgu>/Jwfa (floxed Acan)		EM:10224
Mouse: B6.Cg-Tg(Nes-cre)1Kln/J (Nestin-Cre)	Tronche et al.^49^	JAX 003771

Software and algorithms		

Imaris v9.7.2	Bitplane	https://imaris.oxinst.com
R (4.3.2)	The R Foundation	https://www.r-project.org
R package Seurat (5.1.0)	Hao et al.^94^	https://satijalab.org/seurat/index.html
ggplot2 (3.5.1)	The R Foundation	https://cran.r-project.org/web/packages/ggplot2/index.html
Las X	Leica Microsystems	https://www.leica-microsystems.com/products/microscope-software/p/leicalas-x-ls/
Zen Blue	Zeiss	https://www.zeiss.com/microscopy/us/products/software/zeiss-zen.html
FIJI	Open source	https://imagej.net/ij/ RRID: SCR_003070
Graph Pad Prism v9.0.0	GraphPad Software	https://www.graphpad.com

## Highlights

- Genetic loss of aggrecan disrupts PNNs in the 5xFAD brain
- Aggrecan loss increases amyloid plaque burden
- Microglial clustering around plaques is impaired in the absence of aggrecan
- Spatial transcriptomics indicate altered microglial states with loss of aggrecan and PNNs
