# Supplementary material for: Aggrecan protects against plaque accumulation and is essential for proper microglial responses to plaques
Source: Cell Rep. Author manuscript; Available in PMC 2025 Sep 18. (PMC12445281; doi:10.1016/j.celrep.2025.116064)
Supplement: 1 [file NIHMS2107263-supplement-1.pdf]

Cell Reports, Volume *44*

Supplemental information

Aggrecan protects against plaque accumulation and is essential for proper microglial responses to plaques

Rocio A. Barahona, Nellie E. Kwang, Aashna R. Kono-Soosaipillai, Giovanna Rubio Salgado, Kristine M. Tran, Yueh-Hao Lu, Siddharth Reddy, Celia da Cunha, Eric Velazquez-Rivera, Joshua D. Crapser, Xiangmin Xu, Lindsay A. Hohsfield, and Kim N. Green

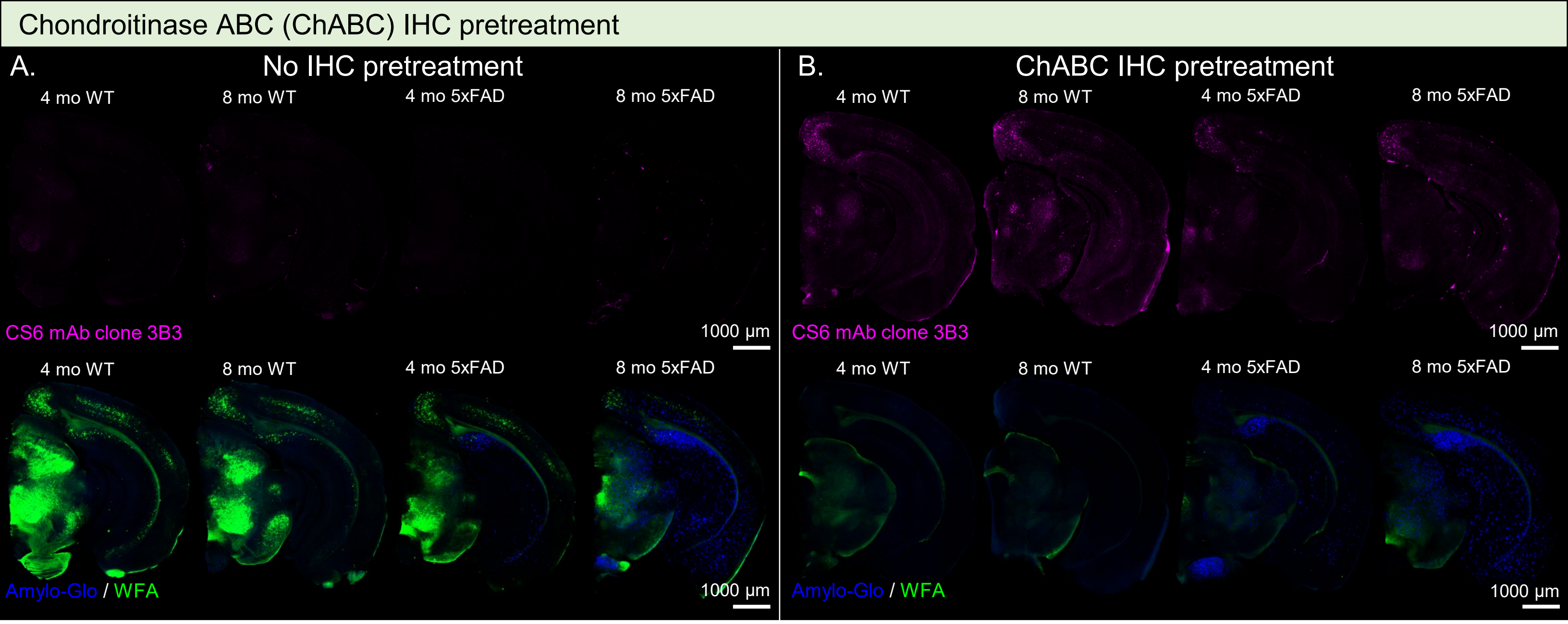

Supplemental Figure 1 – Chondroitinase (ChABC) IHC pretreatment

A) Immunostaining of WT and 5xFAD brain sections in the absence of chABC pretreatment shows lack of CS6 mAb clone 3B3 labeling which recognizes 6-sulfated unsaturated disaccharide neoepitopes generated at the non-reducing terminal of CS-GAG chains that have been pre-digested with chABC. WFA labeling of PNN GAGs remains intact. **B)** Immunostaining of WT and 5xFAD brain sections that underwent a 2-day chABC pretreatment show GAGs were successfully cleaved, evidenced by CS6 mAb clone 3B3 labeling and the lack of WFA signal.

Mouse body weight, cortical thickness, and plasma NfL

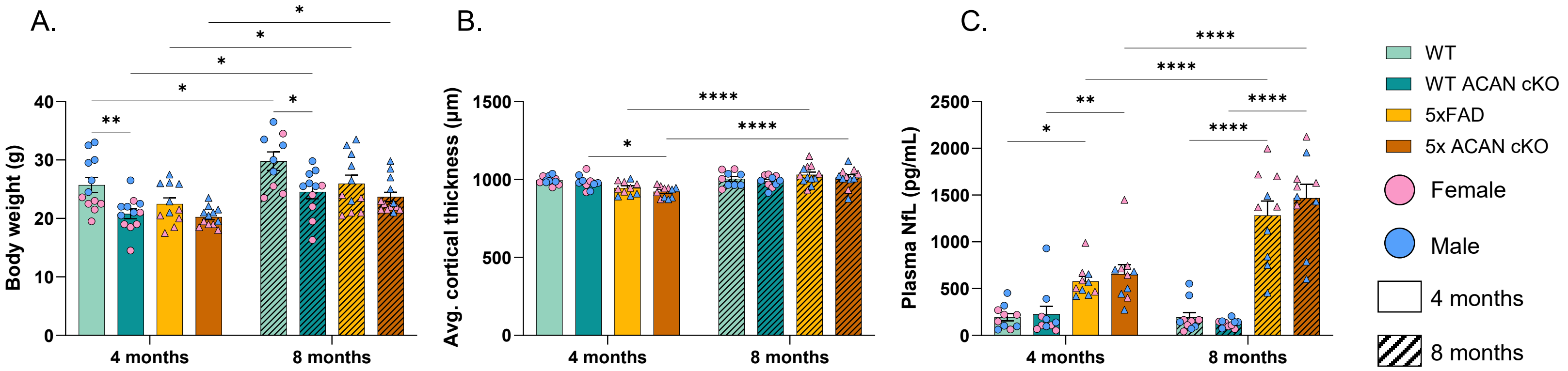

Supplemental Figure 2 – Mouse body weight, cortical thickness, and plasma NfL
A) Total body weight for mice used in the 4-month and 8-month cohort. **B)** Average cortical thickness. **C)** Plasma neurofilament light-chain (NfL) concentration shows expected increases with age and 5xFAD genotype, but no changes between 5xFAD and 5x ACAN cKO. Statistical analysis used a two-way ANOVA with Tukey's multiple comparisons correction. Significance indicated as * p<0.05; ** p<0.01; *** p<0.001. Data are represented as mean ± SEM.

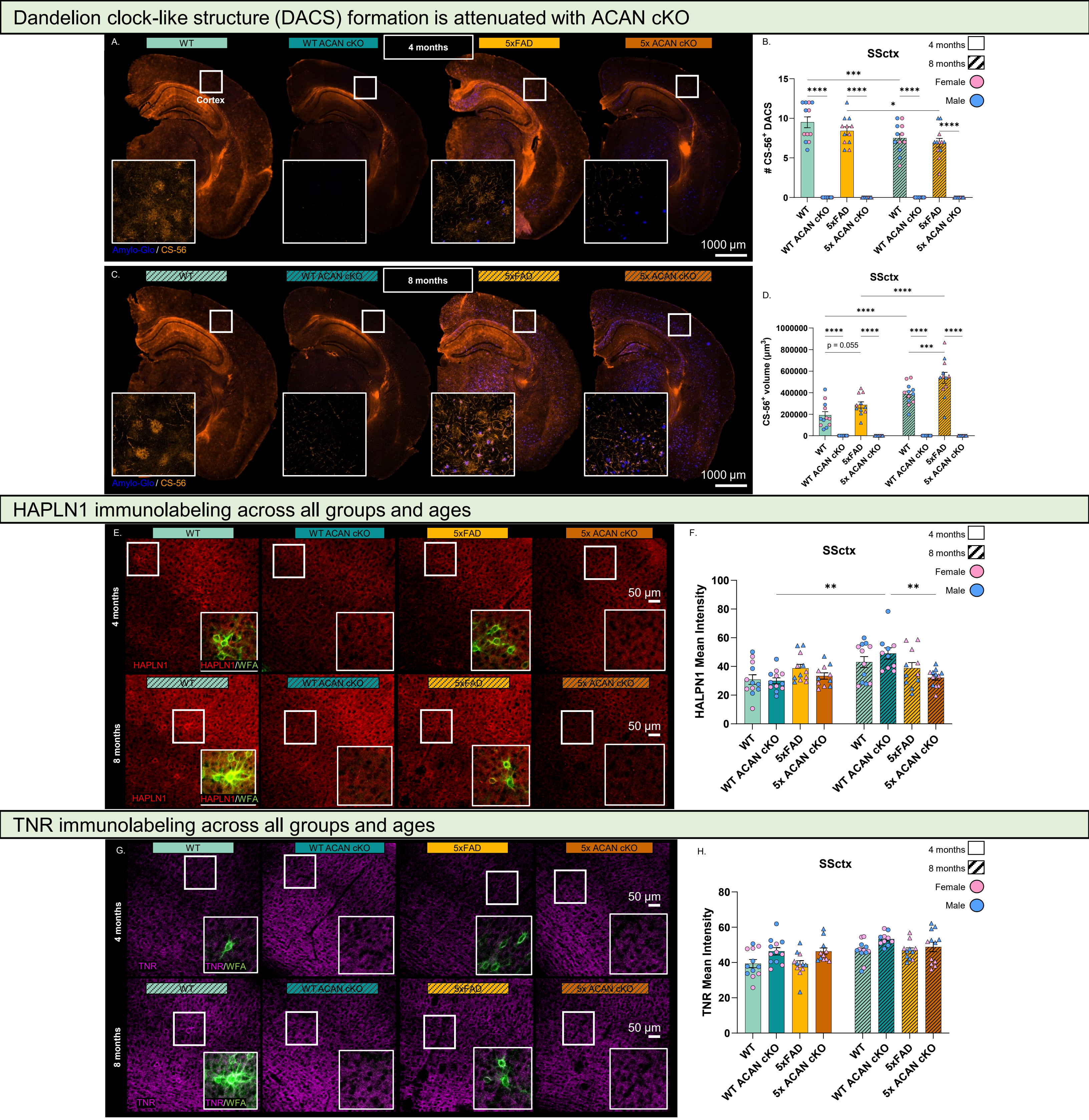

Supplemental Figure 3 – Attenuated DACS formation with ACAN cKO, HAPLN1 and TNR immunolabeling
A) Representative 5x images of all four groups stained for plaques (Amylo-Glo) and anti-chondroitin sulfate antibody CS56 at 4-months and **B)** 8-months with corresponding 20x cortical inset images **C)** Quantification for number of DACS and **D)** CS-56⁺ volume at 4- and 8-months for all four groups. **E)** Representative 20X confocal images of WT, WT ACAN cKO, 5xFAD, and 5x ACAN cKO murine hemibrains at 4- and 8-months stained for HAPLN1 with insets showing colocalization with WFA+ PNNS. **F)** Quantification of HAPLN1 mean intensity at 4- and 8- months for cortical FOVs. **G)** Representative 20X confocal images of WT, WT ACAN cKO, 5xFAD, and 5x ACAN cKO murine hemibrains at 4- and 8-months stained for TNR with insets showing colocalization with WFA+ PNNS. **H)** Quantification of TNR mean intensity at 4- and 8- months for cortical FOVs. Statistical analysis used a two-way ANOVA with Tukey's multiple comparisons correction for CS-56, HAPLN1, and TNR quantifications. Significance indicated as * $p<0.05$; ** $p<0.01$; *** $p<0.001$; ****$p<0.0001$. Data are represented as mean ± SEM.

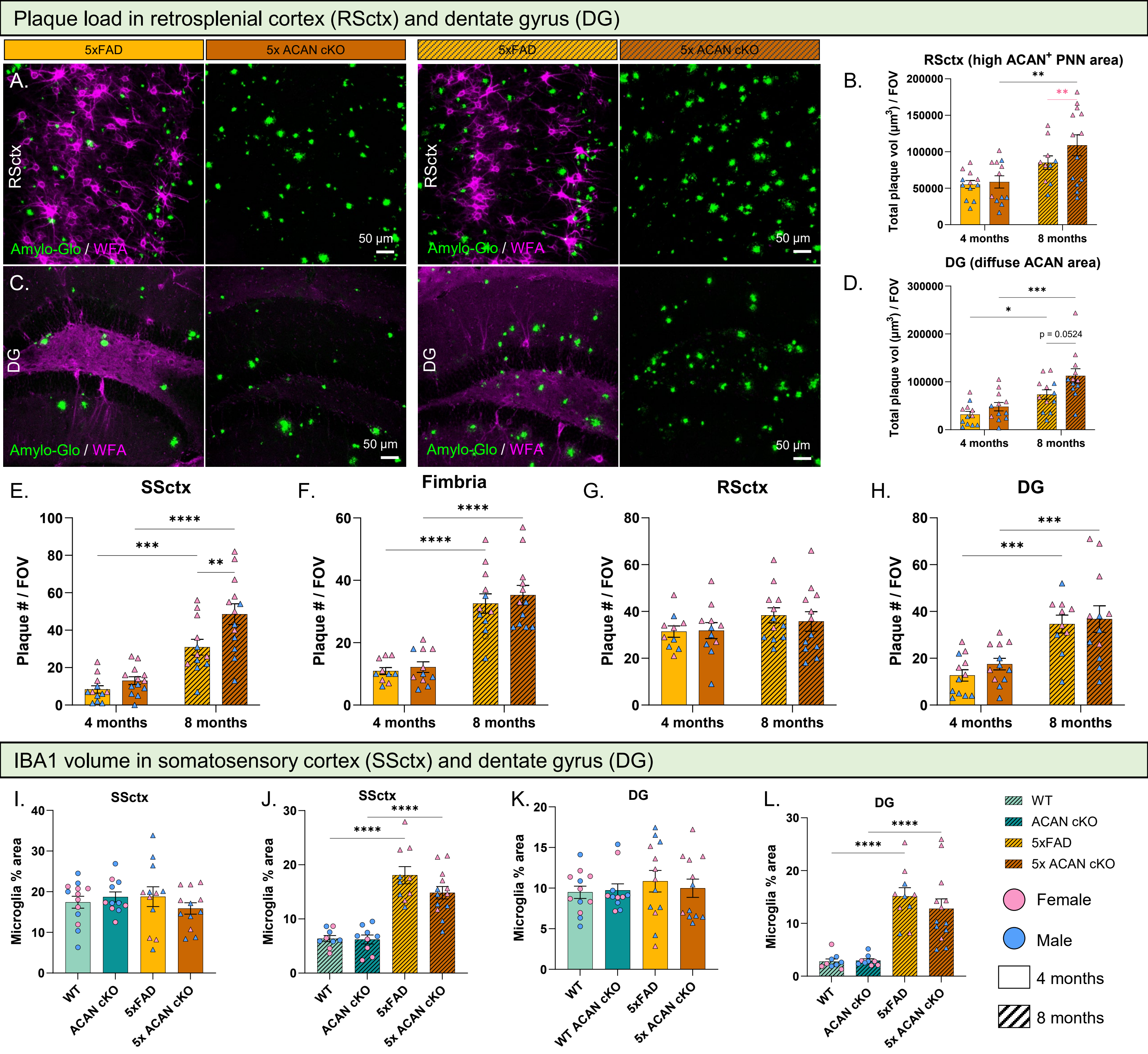

Supplemental Figure 4 – Plaque load, plaque number, and microglia volume

A) Representative 20x confocal images of Amylo-Glo+ plaques and WFA+ PNNs in RSctx of 5xFAD and 5x ACAN cKO at 4- and 8-months with corresponding quantifications for **B)** total plaque volume. **C)** Representative 20x confocal images of Amylo-Glo+ plaques and WFA+ PNNs in DG of 5xFAD and 5x ACAN cKO at 4- and 8-months with corresponding quantifications for **D)** total plaque volume. **E)** Total plaque number per FOV for SSctx, **F)** fimbria, **G)** RSctx, and **H)** DG. **I)** Quantification of SSctx microglia % area in all four groups at 4-months and **J)** 8-months. **K)** Quantification of DG microglia % area in all four groups at 4-months and **L)** 8-months. Statistical analysis used a two-way ANOVA with Tukey's multiple comparisons correction for plaque quantifications and a one-way ANOVA with Tukey's multiple comparisons correction for microglia quantifications. Significance indicated as * p<0.05; ** p<0.01; *** p<0.001; ****p<0.0001. Data are represented as mean ± SEM.

Proinflammatory cytokine concentrations from the soluble fraction of microdissected cortices

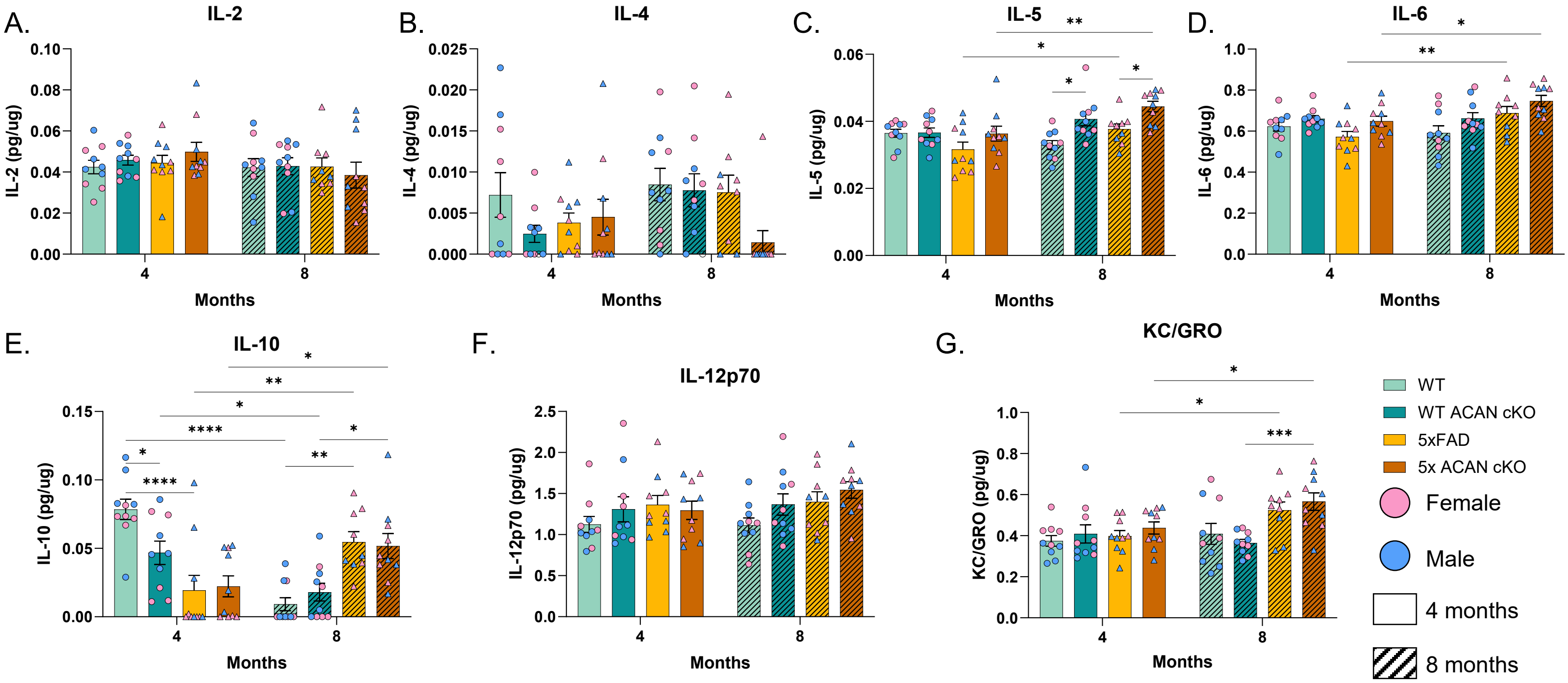

Supplemental Figure 5 – Proinflammatory cytokine concentrations from the soluble fraction of microdissected cortices

A) Quantification of IL-2 **B)** IL-4 **C)** IL-5 **D)** IL-6 **E)** IL-10 **F)** IL-12p70 and **G)** KC/GRO concentration across all genotypes at both 4- and 8-months of age. Statistical analysis used a two-way ANOVA with Tukey's multiple comparisons correction. Significance indicated as * $p<0.05$; ** $p<0.01$; *** $p<0.001$; ****$p<0.0001$. Data are represented as mean ± SEM.

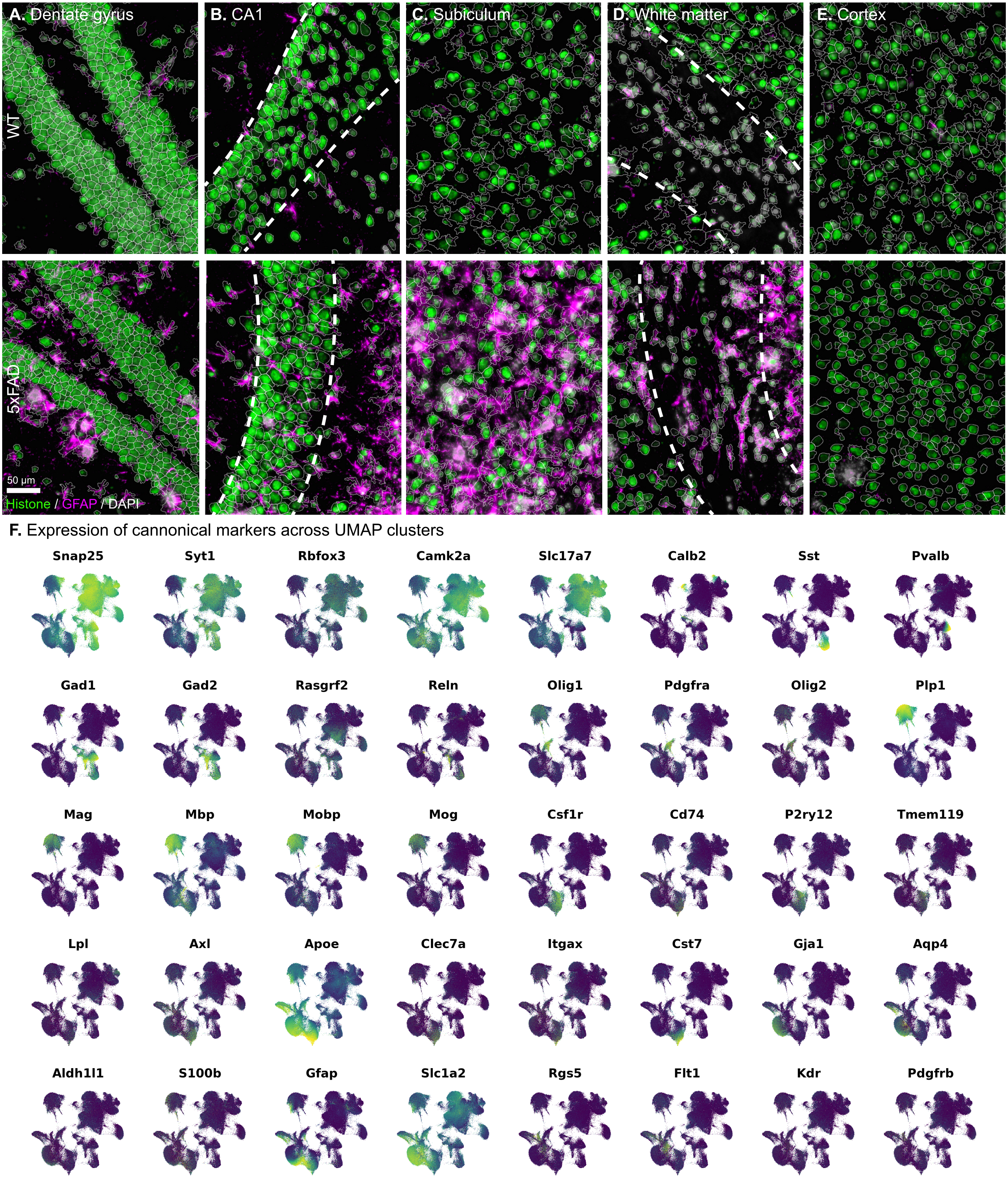

Supplemental Figure 6 – Cell segmentation and gene expression across UMAP clusters

A) Representative images from the AtoMx platform showing cell segmentation for WT and 5xFAD brain tissue labeled with DAPI (white), histone (green), and GFAP (magenta) in the dentate gyrus, **B)** CA1, **C)** subiculum, **D)** white matter, and **E)** cortex. **F)** Expression of canonical markers for different cell types across UMAP clusters.

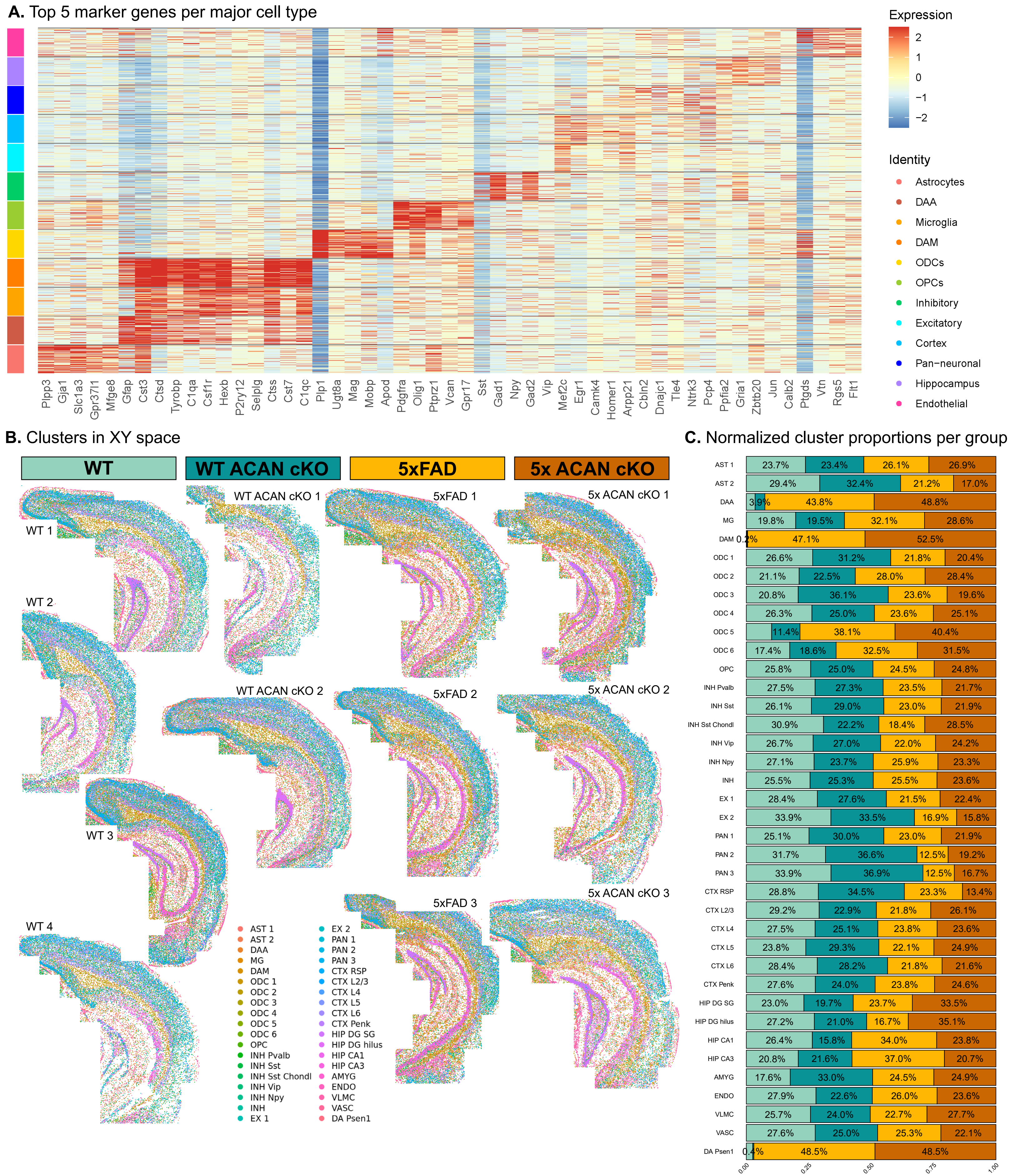

Supplemental Figure 7 – Cell type markers and clusters in XY space

A) A heatmap displaying the top 5 marker genes per major cell type. **B)** All clusters mapped in XY space for all 12 brains used for CosMx experiment. **C)** Stacked histogram displaying normalized proportions of all clusters per group.

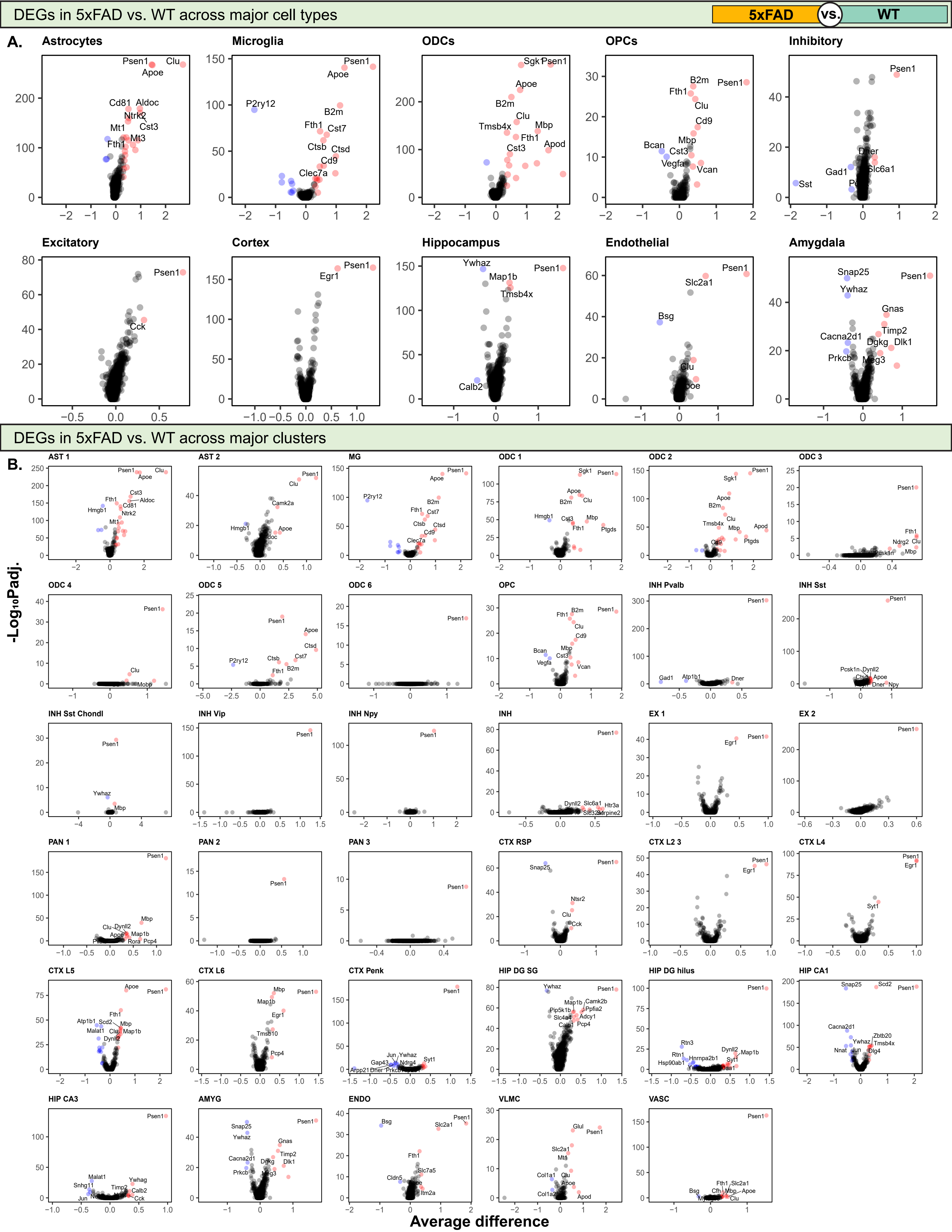

DEGs in 5xFAD vs. WT across major cell types
5xFAD
vs.
WT
A.
Astrocytes
Microglia
ODCs
OPCs
Inhibitory
Excitatory
Cortex
Hippocampus
Endothelial
Amygdala
DEGs in 5xFAD vs. WT across major clusters
B.
AST 1
AST 2
MG
ODC 1
ODC 2
ODC 3
ODC 4
ODC 5
ODC 6
OPC
INH Pvalb
INH Sst
INH Sst Chondl
INH Vip
INH Npy
INH
EX 1
EX 2
PAN 1
PAN 2
PAN 3
CTX RSP
CTX L2 3
CTX L4
CTX L5
CTX L6
CTX Penk
HIP DG SG
HIP DG hilus
HIP CA1
HIP CA3
AMYG
ENDO
VLMC
VASC
-Log₁₀Padj.
Average difference

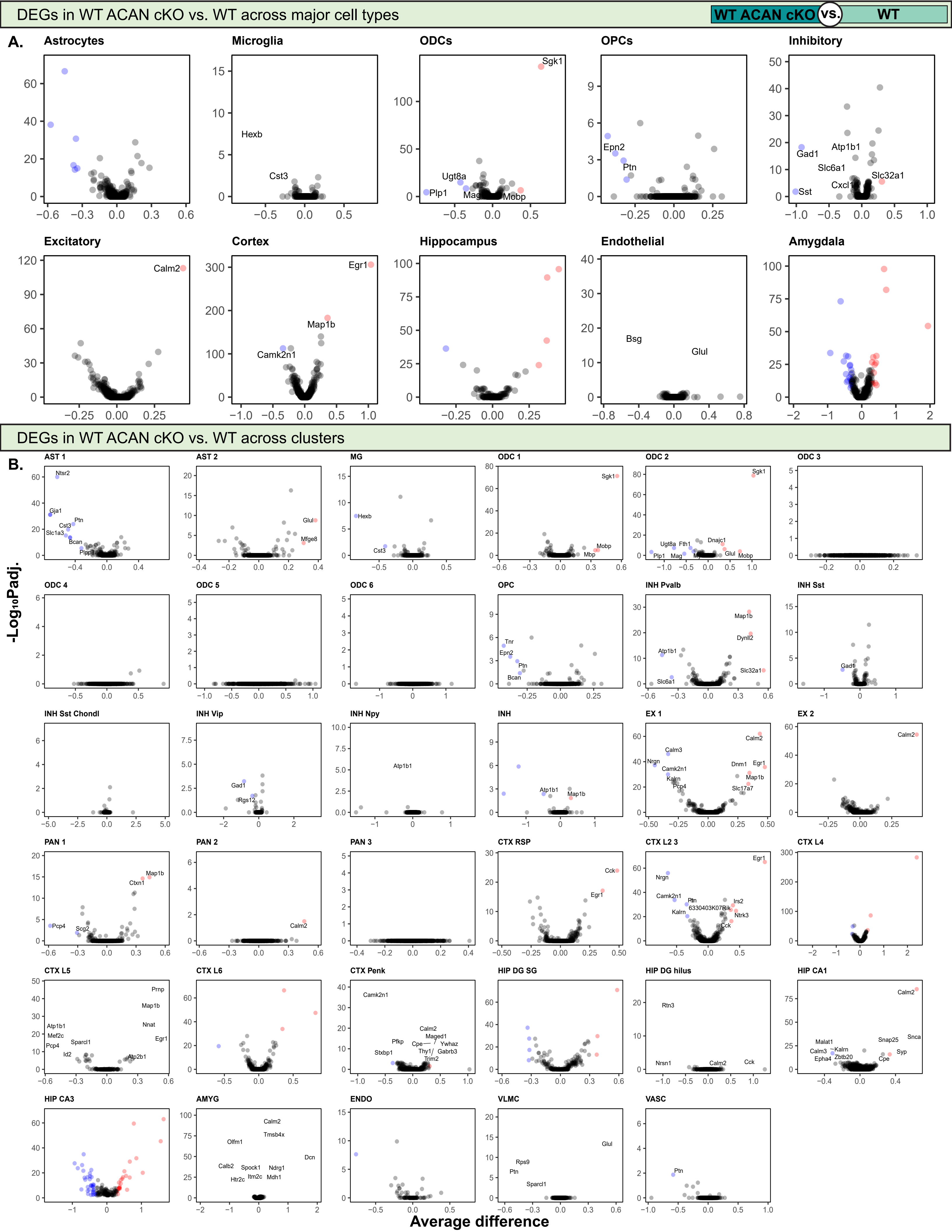

DEGs in WT ACAN cKO vs. WT across major cell types
WT ACAN cKO
vs.
WT
A.
Astrocytes
Microglia
Hexb
Cst3
ODCs
Sgk1
Ugt8a
Plp1
Mag
Mobp
OPCs
Epn2
Ptn
Inhibitory
Gad1
Atp1b1
Slc6a1
Cxcl1
Slc32a1
Sst
Excitatory
Calm2
Cortex
Egr1
Map1b
Camk2n1
Hippocampus
Endothelial
Bsg
Glul
Amygdala
DEGs in WT ACAN cKO vs. WT across clusters
B.
AST 1
Ntsr2
Gja1
Ptn
Cst3
Slc1a3
Bcan
Plpp3
AST 2
Glul
Mfge8
MG
Hexb
Cst3
ODC 1
Sgk1
Mobp
Mbp
ODC 2
Sgk1
Ugt8a
Fth1
Dnajc1
Plp1
Mag
Glul
Mobp
ODC 3
ODC 4
ODC 5
ODC 6
OPC
Tnr
Epn2
Ptn
Bcan
INH Pvalb
Map1b
Dynll2
Atp1b1
Slc32a1
Slc6a1
INH Sst
Gad1
INH Sst Chondl
INH Vip
Gad1
Rgs12
INH Npy
Atp1b1
INH
Atp1b1
Map1b
EX 1
Calm2
Calm3
Nrgn
Dnm1
Egr1
Camk2n1
Kalrn
Map1b
Pcp4
Slc17a7
EX 2
Calm2
PAN 1
Map1b
Ctxn1
Pcp4
Scg2
PAN 2
Calm2
PAN 3
CTX RSP
Cck
Egr1
CTX L2 3
Egr1
Nrgn
Camk2n1
Ptn
Irs2
6330403K07Rik
Kalrn
Ntrk3
Cck
CTX L4
CTX L5
Prnp
Map1b
Atp1b1
Nnat
Mef2c
Egr1
Sparcl1
Pcp4
Id2
Atp2b1
CTX L6
CTX Penk
Camk2n1
Calm2
Maged1
Pfkp
Cpe
Ywhaz
Thy1
Gabrb3
Stxbp1
Trim2
HIP DG SG
HIP DG hilus
Rtn3
Nrsn1
Calm2
Cck
HIP CA1
Calm2
Malat1
Snap25
Snca
Calm3
Kalrn
Syp
Epha4
Zbtb20
Cpe
HIP CA3
AMYG
Calm2
Tmsb4x
Olfm1
Dcn
Calb2
Spock1
Ndrg1
Htr2c
Itm2c
Mdh1
ENDO
VLMC
Glul
Rps9
Ptn
Sparcl1
VASC
Ptn
-Log₁₀Padj.
Average difference

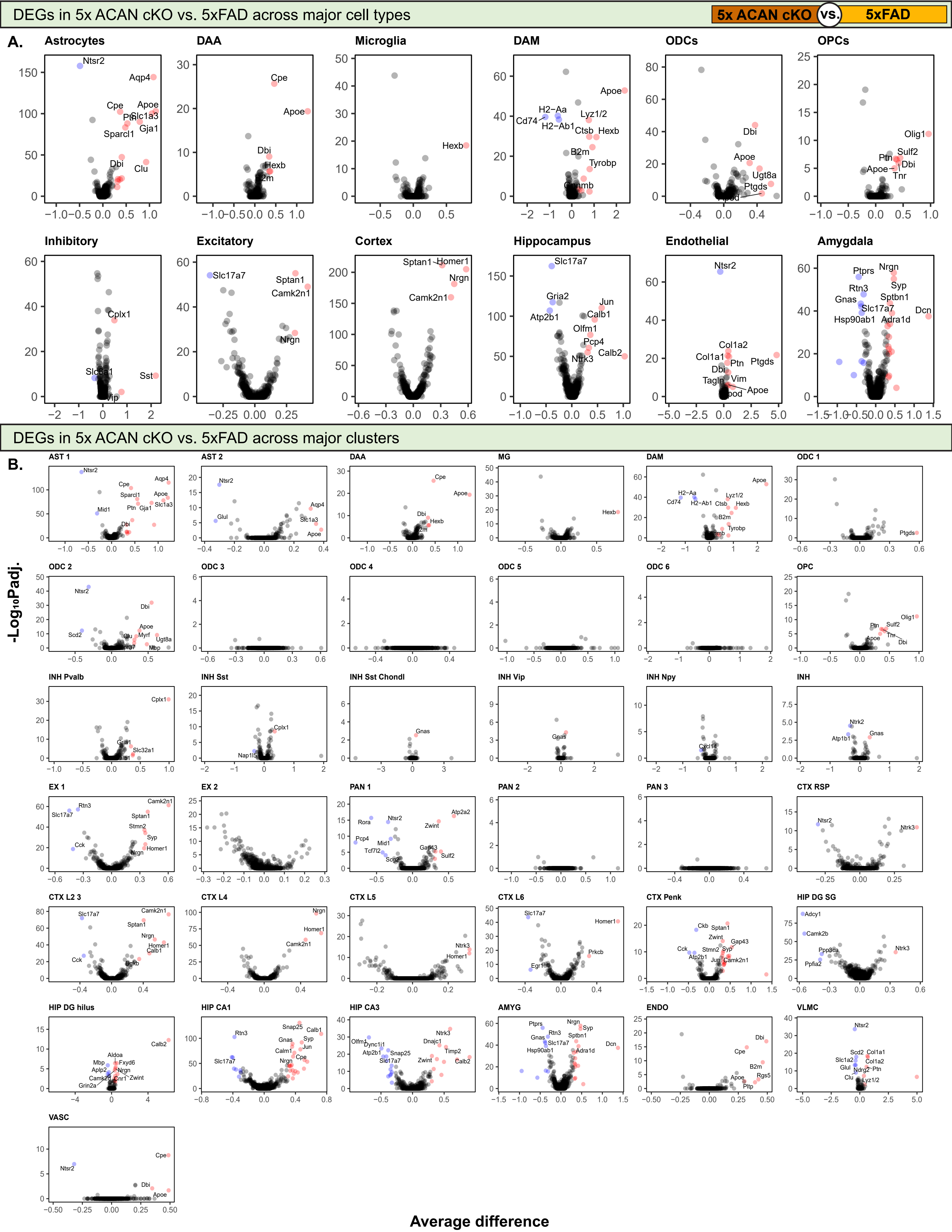
DEGs in 5x ACAN cKO vs. 5xFAD across major cell types
5x ACAN cKO
vs.
5xFAD
A.
Astrocytes
DAA
Microglia
DAM
ODCs
OPCs
Inhibitory
Excitatory
Cortex
Hippocampus
Endothelial
Amygdala
Ntsr2
Aqp4
Cpe
Apoe
Ptn
Slc1a3
Gja1
Sparcl1
Dbi
Clu
Hexb
B2m
H2-Aa
Cd74
H2-Ab1
Lyz1/2
Ctsb
Tyrobp
Gpnmb
Ugt8a
Ptgds
Apod
Olig1
Sulf2
Tnr
Cplx1
Slc6a1
Sst
Vip
Slc17a7
Sptan1
Camk2n1
Nrgn
Homer1
Gria2
Jun
Atp2b1
Calb1
Olfm1
Pcp4
Calb2
Ntrk3
Col1a2
Col1a1
Tagln
Vim
Ptprs
Rtn3
Syp
Gnas
Sptbn1
Hsp90ab1
Adra1d
Dcn
DEGs in 5x ACAN cKO vs. 5xFAD across major clusters
B.
AST 1
AST 2
DAA
MG
DAM
ODC 1
ODC 2
ODC 3
ODC 4
ODC 5
ODC 6
OPC
INH Pvalb
INH Sst
INH Sst Chondl
INH Vip
INH Npy
INH
EX 1
EX 2
PAN 1
PAN 2
PAN 3
CTX RSP
CTX L2 3
CTX L4
CTX L5
CTX L6
CTX Penk
HIP DG SG
HIP DG hilus
HIP CA1
HIP CA3
AMYG
ENDO
VLMC
VASC
-Log₁₀Padj.
Average difference

Supplemental Figure 8 – DEGs in 5xFAD vs. WT

A) DEGs in 5xFAD vs. WT for all major cell types and **B)** individual clusters.

Supplemental Figure 9 – DEGs in WT ACAN cKO vs. WT

A) DEGs in WT ACAN cKO vs. WT for all major cell types and **B)** individual clusters.

Supplemental Figure 10 – DEGs in 5x ACAN cKO vs. 5xFAD

A) DEGs in 5x ACAN cKO vs. 5xFAD for all major cell types and **B)** individual clusters

Average gene expression per brain per cell type of ECM-related genes

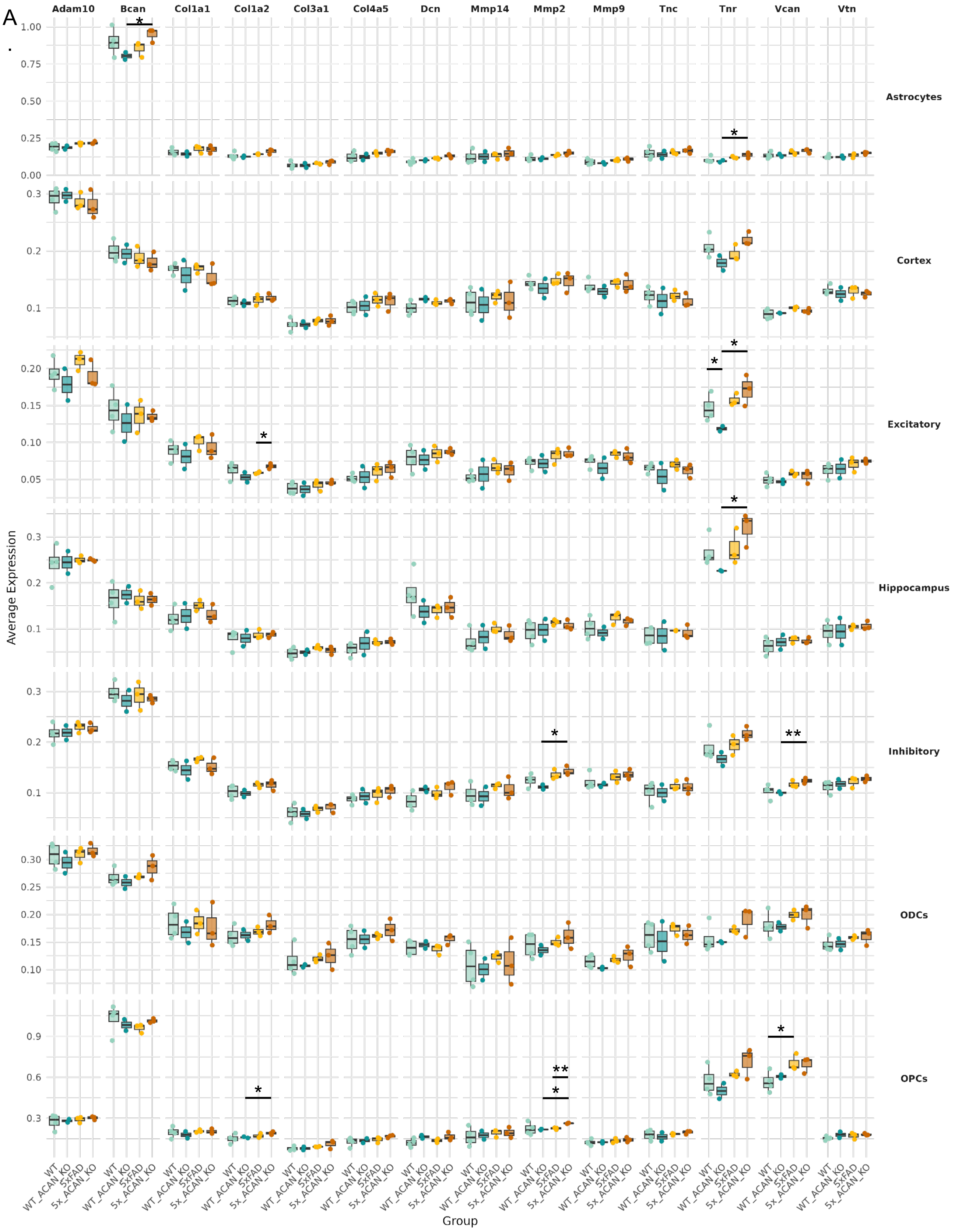

Supplemental Figure 11 – Average gene expression of ECM-related genes per brain per cell type across groups

A) Box plots represent the distribution of average gene expression levels for each experimental group. Data are stratified by gene of interest and the following major cell types/brain regions: Astrocytes, Cortex, Excitatory, Hippocampus, Inhibitory, ODCs, and OPCs. The y-axis represents average gene expression, and the x-axis displays experimental groups. Statistical analysis used a one-way ANOVA with Tukey's multiple comparisons correction. Significance indicated as * p<0.05; ** p<0.01; *** p<0.001.

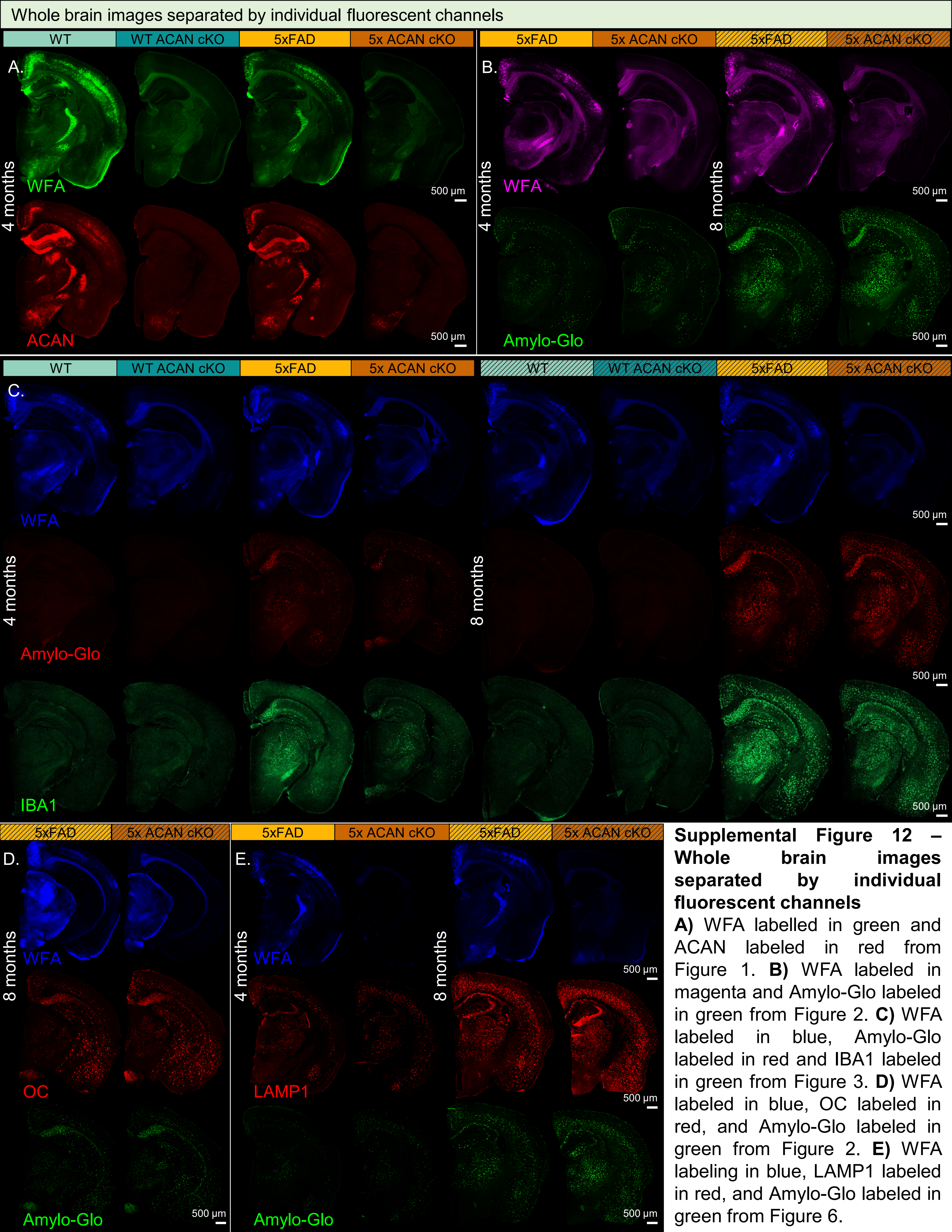

Supplemental Figure 12 – Whole brain images separated by individual fluorescent channels

A) WFA labelled in green and ACAN labeled in red from Figure 1. **B)** WFA labeled in magenta and Amylo-Glo labeled in green from Figure 2. **C)** WFA labeled in blue, Amylo-Glo labeled in red and IBA1 labeled in green from Figure 3. **D)** WFA labeled in blue, OC labeled in red, and Amylo-Glo labeled in green from Figure 2. **E)** WFA labeling in blue, LAMP1 labeled in red, and Amylo-Glo labeled in green from Figure 6.
